# Ecology, Not Host Phylogeny, Shapes the Oral Microbiome in Closely Related Species

**DOI:** 10.1093/molbev/msac263

**Published:** 2022-12-06

**Authors:** Markella Moraitou, Adrian Forsythe, James A Fellows Yates, Jaelle C Brealey, Christina Warinner, Katerina Guschanski

**Affiliations:** Animal Ecology, Department of Ecology and Genetics, Uppsala University, 75236 Uppsala, Sweden; Institute of Ecology and Evolution, School of Biological Sciences, University of Edinburgh, Edinburgh EH9 3FL, United Kingdom; Animal Ecology, Department of Ecology and Genetics, Uppsala University, 75236 Uppsala, Sweden; Department of Archaeogenetics, Max Planck Institute for Evolutionary Anthropology, 04103 Leipzig, Germany; Department of Paleobiotechnology, Leibniz Institute for Natural Product Research and Infection Biology Hans Knöll Institute, 07745 Jena, Germany; Department of Natural History, NTNU University Museum, Norwegian University of Science and Technology, 7491 Trondheim, Norway; Department of Archaeogenetics, Max Planck Institute for Evolutionary Anthropology, 04103 Leipzig, Germany; Department of Paleobiotechnology, Leibniz Institute for Natural Product Research and Infection Biology Hans Knöll Institute, 07745 Jena, Germany; Faculty of Biological Sciences, Friedrich Schiller University, 07743 Jena, Germany; Department of Anthropology, Harvard University, Cambridge, MA 02138, USA; Animal Ecology, Department of Ecology and Genetics, Uppsala University, 75236 Uppsala, Sweden; Institute of Ecology and Evolution, School of Biological Sciences, University of Edinburgh, Edinburgh EH9 3FL, United Kingdom; Science for Life Laboratory, 75237 Uppsala, Sweden

**Keywords:** ancient DNA, dental calculus, museum specimens, gorilla, metagenome-assembled genomes, shotgun metagenomics

## Abstract

Host-associated microbiomes are essential for a multitude of biological processes. Placed at the contact zone between external and internal environments, the little-studied oral microbiome has important roles in host physiology and health. Here, we investigate the roles of host evolutionary relationships and ecology in shaping the oral microbiome in three closely related gorilla subspecies (mountain, Grauer's, and western lowland gorillas) using shotgun metagenomics of 46 museum-preserved dental calculus samples. We find that the oral microbiomes of mountain gorillas are functionally and taxonomically distinct from the other two subspecies, despite close evolutionary relationships and geographic proximity with Grauer's gorillas. Grauer's gorillas show intermediate bacterial taxonomic and functional, and dietary profiles. Altitudinal differences in gorilla subspecies ranges appear to explain these patterns, suggesting a close connection between dental calculus microbiomes and the environment, likely mediated through diet. This is further supported by the presence of gorilla subspecies-specific phyllosphere/rhizosphere taxa in the oral microbiome. Mountain gorillas show a high abundance of nitrate-reducing oral taxa, which may promote adaptation to a high-altitude lifestyle by modulating blood pressure. Our results suggest that ecology, rather than evolutionary relationships and geographic distribution, shape the oral microbiome in these closely related species.

## Introduction

The microbial communities found on and inside multicellular organisms are not only remarkably diverse, but also play a crucial role in important biological processes, such as energy uptake (Arora and Sharma 2011; Nieuwdorp et al. 2014), detoxification (Nichols et al. 2019; Turner and Bucking 2019), immune responses (West et al. 2015; Nikitakis et al. 2017), and even neurochemical and hormonal processes that eventually influence behavior (Suzuki 2017; Vuong et al. 2017). Although most studies have focused on the gut microbiome, the oral microbiome is also of great interest, as it connects the external and the internal environments and is located directly at the entry point to the digestive and respiratory tracts. The oral microbiome plays an important role in oral diseases, such as dental caries and periodontitis, and has been implicated in systemic disorders, including cardiovascular disease (Nakano et al. 2011), atherosclerosis (Teles and Wang 2011), Alzheimer's disease (Olsen and Singhrao 2015), several cancers (Flynn et al. 2016; Gao et al. 2016), and preterm births (Cobb et al. 2017).

Multi-species assemblages, such as the gut microbiome and also parasite communities, are primarily influenced by host ecology and evolutionary relationships (Poulin 1995; Ley et al. 2008; Muegge et al. 2011; Nishida and Ochman 2018, 2019; Beer et al. 2019; Youngblut et al. 2019; Schneider-Crease 2020; Weinstein et al. 2021). However, the relative importance and effect size of these factors differ from system to system. It is also unclear which of the two predominantly drives the evolution of the oral microbiome and how they interact. Early studies suggested that the oral microbiome is strongly heritable and mostly transferred vertically from the mother to offspring (Corby et al. 2007; Li et al. 2007; Dominguez-Bello et al. 2010). Studies of wild animals have revealed species-specific oral microbiome communities (Brealey et al. 2020; Ozga and Ottoni 2021). Both lines of evidence suggest that host evolutionary relationships have a strong effect on oral microbiome structure. However, other studies have highlighted the effects of diet on the oral microbiome (Wade 2013; Hyde, Luk, et al. 2014; Adler et al. 2016; Nearing et al. 2020; Janiak et al. 2021), including during maturation, as the diet changes from infancy to adulthood (Cephas et al. 2011). Yet, distinguishing between host evolutionary and ecological factors as well as their contribution to oral microbiome evolution remains difficult, as most previous studies have focused on rather distantly related host species that occupy distinct ecological niches (Li et al. 2013; Soares-Castro et al. 2019; Boehlke et al. 2020; Smith et al. 2021).

In recent years, dental calculus—the calcified form of dental plaque that forms on the teeth of mammals—has emerged as a useful material for the comparative study of oral microbiomes in diverse mammalian species (Ottoni et al. 2019; Brealey et al. 2020; Fellows Yates et al. 2021). Since dental plaque undergoes periodic mineralizations, it effectively fossilizes on the living host, reducing postmortem contamination (Warinner et al. 2015). It thus preserves a snapshot of oral and respiratory microbial communities (Warinner et al. 2014), dietary components (Adler et al. 2013; Radini et al. 2017; Sawafuji et al. 2020), and host DNA (Mann et al. 2018). Museum collections can be efficiently used for the study of oral microbiomes from wild animals (Brealey et al. 2020; Fellows Yates et al. 2021), minimizing the exposure and disturbances associated with sampling from live hosts. Furthermore, the detection of damage patterns, a common verification method for ancient DNA (Briggs et al. 2007), can also be implemented on such historical specimens to distinguish endogenous taxa from modern contaminants. Previous research suggests that microbial communities in historical and modern dental calculus are remarkably similar (Velsko et al. 2019); therefore, studying museum specimens provides reliable information about present-day microbiomes. The exceptional preservation in the calcified matrix also allows the assembly of near complete metagenome-assembled genomes (MAGs) (Brealey et al. 2020; Fotakis et al. 2020).

**Fig. 1.**
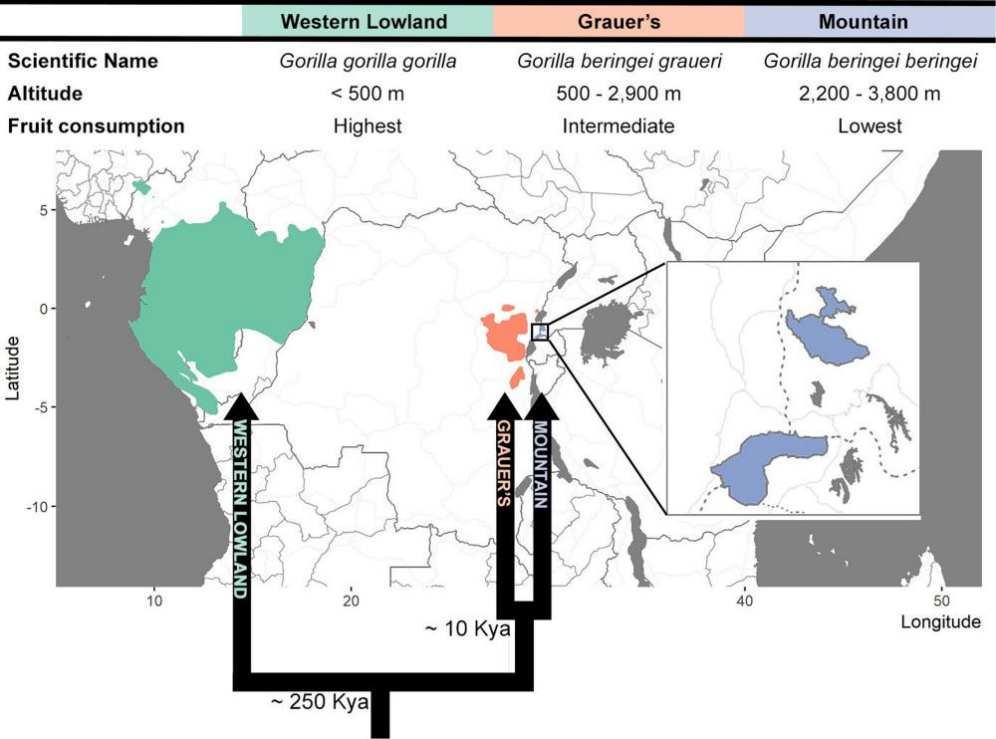
A summary of geographic distributions, phylogenetic relationships, and main ecological differences between the gorilla subspecies studied here. Taxon ranges are adapted from IUCN (IUCN 2022). Approximate divergence times for eastern and western gorillas (McManus et al. 2015; Xue et al. 2015) and the eastern subspecies, mountain and Grauer's gorillas (Roy et al. 2014), are included below the nodes in the phylogeny.

In this study, we use dental calculus to uncover ecological and evolutionary factors that drive oral microbiome evolution in a group of closely related species. We focus on three gorilla subspecies: western lowland gorilla (*G. g. gorilla*), and two eastern subspecies Grauer's gorilla (*G. b. graueri*) and mountain gorilla (*G. b. beringei;*fig. 1). Western lowland gorillas diverged from eastern gorillas approximately 250,000 years ago (McManus et al. 2015; Xue et al. 2015) and are geographically separated from them by the Congo Basin. Mountain and Grauer's gorillas diverged from each other ca. 10,000 years ago (Roy et al. 2014). While all gorillas are folivores, the habitats of the three subspecies differ in altitude, which leads to substantial differences in diet. This is best exemplified by the degree of frugivory, as the availability of fruit decreases with altitude (Harcourt and Stewart 2007). Western lowland gorillas occupy elevations below 500 metres above sea level (masl), where fruit, albeit seasonal, is abundant (Doran et al. 2002; Rogers et al. 2004). Grauer's gorillas occupy the largest altitudinal range of all gorillas (500 to 2,900 masl) (Plumptre et al. 2016), and therefore, low- and high-altitude populations differ in the amount of fruits they consume (Doran et al. 2002; Ganas et al. 2004; Rogers et al. 2004; van der Hoek et al. 2021; Michel et al. 2022). Mountain gorillas are found at even higher elevations, with most of the range of the Virunga Massif population above 2,200 masl and up to 3,800 masl. They primarily rely on herbaceous vegetation with only occasional fruit consumption (Ganas et al. 2004; Yamagiwa et al. 2005). Gorillas at low altitudes also consume a greater diversity of plants compared with high-altitude populations (Doran et al. 2002; Ganas et al. 2004; Rogers et al. 2004; van der Hoek et al. 2021; Michel et al. 2022).

The ecological differences that exist between the closely related gorilla subspecies provide an opportunity to evaluate the effects of host evolutionary relationships and ecological factors on oral microbial communities. We predict that if host phylogeny is more important than ecology, we will detect a greater taxonomic, and possibly functional, similarity between Grauer's and mountain gorillas, than with western lowland gorillas. Conversely, if ecology is the primary factor structuring the oral microbiome, we will observe an association between microbiome composition and ecological variables independent of host phylogeny. Since altitude is a simple metric that affects multiple aspects of the gorilla habitat and diet, we used it as a proxy for ecology in our analyses. Following a shotgun metagenomic sequencing of dental calculus samples from museum specimens, we find that ecology outweighs phylogeny and has a large impact on the taxonomic composition and functional capacities of the oral microbiome. We highlight subspecies-specific differences that can be attributed to ecological (likely dietary) factors and identify oral bacteria that are specifically enriched in mountain gorillas and may facilitate their adaptation to the high-altitude environment.

## Results

### Data Preprocessing and Confirmation of Oral Microbial Signature

All samples in this study were collected from gorilla skulls preserved in natural history collections. We generated shotgun sequencing data for 26 samples of gorilla dental calculus (Methods, supplementary table S1, Supplementary Material online) and combined it with two gorilla dental calculus metagenomes that were previously published by Brealey et al. (2020). This set of 28 samples was processed and sequenced with the same methods and in the same sequencing facility and, therefore, for simplicity, we refer to it as the “newly generated” dataset. We have also obtained published data from 29 gorilla dental calculus metagenomes from Fellows Yates et al. (2021), which we refer to as “Fellows Yates et al. 2021”. In total, we analyzed paired-end shotgun sequencing data from 57 gorilla dental calculus samples (supplementary table S1, Supplementary Material online). Material from four dental calculus samples was split and processed independently by us and Fellows Yates et al. (2021), producing technical replicates that allowed us to assess potential dataset-specific differences. Only the replicate with the largest number of reads for each sample pair was retained for further analyses.

Sequencing produced a mean of 15,533,319 reads per sample (1,032–95,367,058 reads; supplementary table S1, Supplementary Material online) and 105,653 reads per negative control (both extraction and library preparation, 19–1,132,867 reads; supplementary table S1, Supplementary Material online). After preprocessing (which included removal of poly-G tails, adapter and barcode sequences, merging of forward and reverse reads, and quality filtering), *phiX*, gorilla, and human sequences were removed by mapping against respective reference genomes. The resulting unmapped reads were used for taxonomic classification using Kraken2 (Wood et al. 2019) with the standard database, which includes all bacterial, archaeal, and viral genomes from NCBI. Samples with low read counts, low proportions of oral taxa, and one of each technical duplicate sample (with the lowest number of reads), were removed (Methods). The final dataset consisted of 46 dental calculus samples (13 western lowland gorillas, 17 mountain gorillas, and 16 Grauer's gorillas; supplementary fig. S1, supplementary table S2, Supplementary Material online), each containing 561,978–77,307,443 reads (mean: 11,365,074, SD: 15,577,988; supplementary table S1, Supplementary Material online). We then applied a multi-step decontamination procedure, relying on negative controls and museum environmental samples (Methods). We retained six genera that have been listed as common contaminants by Salter et al. (2014) and Weyrich et al. (2019), but contained known oral taxa (Chen et al. 2010; Fellows Yates et al. 2021), including *Streptococcus oralis* and *Staphylococcus saprophyticus.* Species belonging to these genera were retained after confirming, where possible, that they exhibit typical postmortem DNA damage patterns (Methods). The final dataset contained a total of 1,007 microbial species (in 430 genera), of which 3.4% (*n* = 34) were members of the core hominid oral microbiome (Fellows Yates et al. 2021) and 4.2% (*n* = 42) members of the Human Oral Microbiome Database (Chen et al. 2010). These microbial species accounted for ca. 14% of the total microbial abundance.

### Oral Microbiome Diversity and Composition Differ by Host Subspecies

To evaluate the effect of host evolutionary relationships and host sex on oral microbiome composition and function, we performed molecular host subspecies and sex assignments (Supplementary material online). Subspecies assignments agreed with museum records (supplementary table S2, Supplementary Material online), whereas molecular sex assignments showed several discrepancies with museum records (supplementary table S1, Supplementary Material online). Therefore, all analyses testing for the effect of host sex were performed twice, using either the molecular or museum sex assignments.

**Fig. 2.**
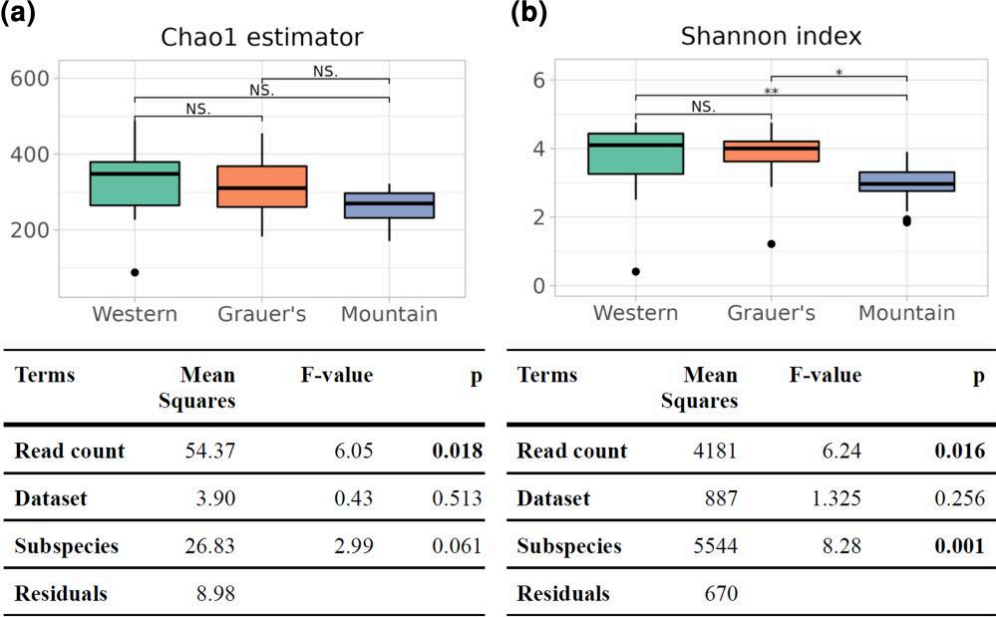
(*a*) Estimates of community richness, using Chao1 index and (*b*) community evenness, using Shannon index, alongside corresponding ANOVA results (tables). Pairwise comparisons in (*a*) and (*b*) show FDR-adjusted *P* values of Tukey's test (NS, not significant, *P < 0.05, **P < 0.01). For the ANOVA models, sqrt(500-x) and exp(*x*) transformations were implemented on the Chao1 estimator and Shannon index values, respectively.

We evaluated the differences in microbial alpha diversity between gorilla subspecies by measuring species richness and community evenness (fig. 2). Normalized microbial richness did not differ among gorilla subspecies (ANOVA, *P* = 0.061; fig. 2*a*); however, mountain gorillas had significantly lower evenness than the other subspecies (ANOVA, *P* < 0.001; fig. 2*b*).

**Fig. 3.**
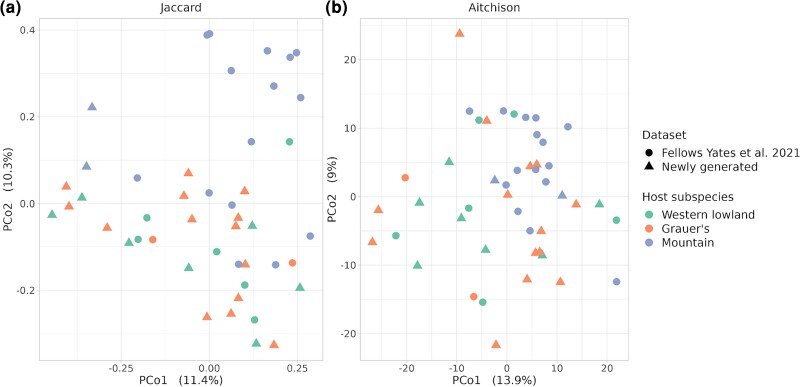
Principal coordinate analysis (PCoA) plots of the individual microbiomes based on (*a*) Jaccard distance and (*b*) Aitchison distance. Host subspecies are displayed in different colors, whereas the dataset is indicated by different shapes (circles for Fellows Yates et al. (2021) and triangles for the newly generated data).

We find significant differences in microbiome composition between gorilla subspecies (fig. 3 and table 1). This effect persisted even after other potentially confounding factors, such as sequencing depth and dataset, were taken into account. We confirmed that belonging to different datasets had little effect on relative abundance by considering three pairs of samples that were derived from the same museum specimen, but were independently processed in this study and in Fellows Yates et al. (2021). Duplicate samples appeared close to each other on ordination plots (supplementary fig. S2, Supplementary Material online). The fourth sample pair (G0004-IBA002) was excluded, as both replicates had lower than 3% oral component (supplementary fig. S3, Supplementary Material online). Pairwise PERMANOVAs (PERMutational ANalysis Of VAriance) (Anderson 2001) showed strong differentiation between mountain gorillas and the other two subspecies, using both presence–absence (Jaccard) and abundance metrics (Aitchison) (table 1). Lastly, we found no significant effect of individual age or sex on the oral microbiome composition (supplementary table S3, Supplementary Material online).

**Table 1.** Effects of Dataset and Host Subspecies on Microbiome Composition.

Terms	Within-factor Comparison	Jaccard Distances		Aitchison Distances	
		*R* ^2^	*P*	*R* ^2^	*P*
Read count		0.069	**<0.001**	0.023	0.297
Dataset		0.037	**0**.**005**	0.029	0.083
Subspecies		0.067	**<0**.**001**	0.074	**0**.**001**
	Western lowland versus Grauer's	0.029	0.853	0.041	0.223
	Western lowland versus mountain	0.086	**0**.**001**	0.085	**0**.**001**
	Grauer's versus mountain	0.073	**0.001**	0.072	**0.001**
Residuals		0.826	-	0.874	-

PERMANOVA results, showing the effect size (*R*^2^) and *P* value of factors putatively contributing to the variance among sampled microbiota, using Jaccard (reflecting presence–absence of taxa) and Aitchison (reflecting relative abundance of taxa) distances. Pairwise PERMANOVA between subspecies shows the effect size (*R*^2^) and *P* values adjusted for false discovery rate. *P* values below 0.05 are shown in bold.

Among the 1,007 microbial (species-level) taxa in our dataset, we detected 91 that significantly differed in abundance among gorilla subspecies and 11 that significantly differed in abundance between the datasets (supplementary table S4, Supplementary Material online). Ten of these 11 taxa also differed in abundance by subspecies but because they could reflect dataset-specific artifacts, we removed all species belonging to these genera from the list of subspecies-associated taxa. Among the remaining 78 differentially abundant taxa, all but six were absent from at least one host subspecies. The abundances of these taxa were comparable to taxa that did not differ in abundance between subspecies (*n* = 929; *t*-test: *t*[123.59] = 1.24, *P* = 0.22), suggesting that their absence from some host subspecies is unlikely to be due to low abundance.

**Fig. 4.**
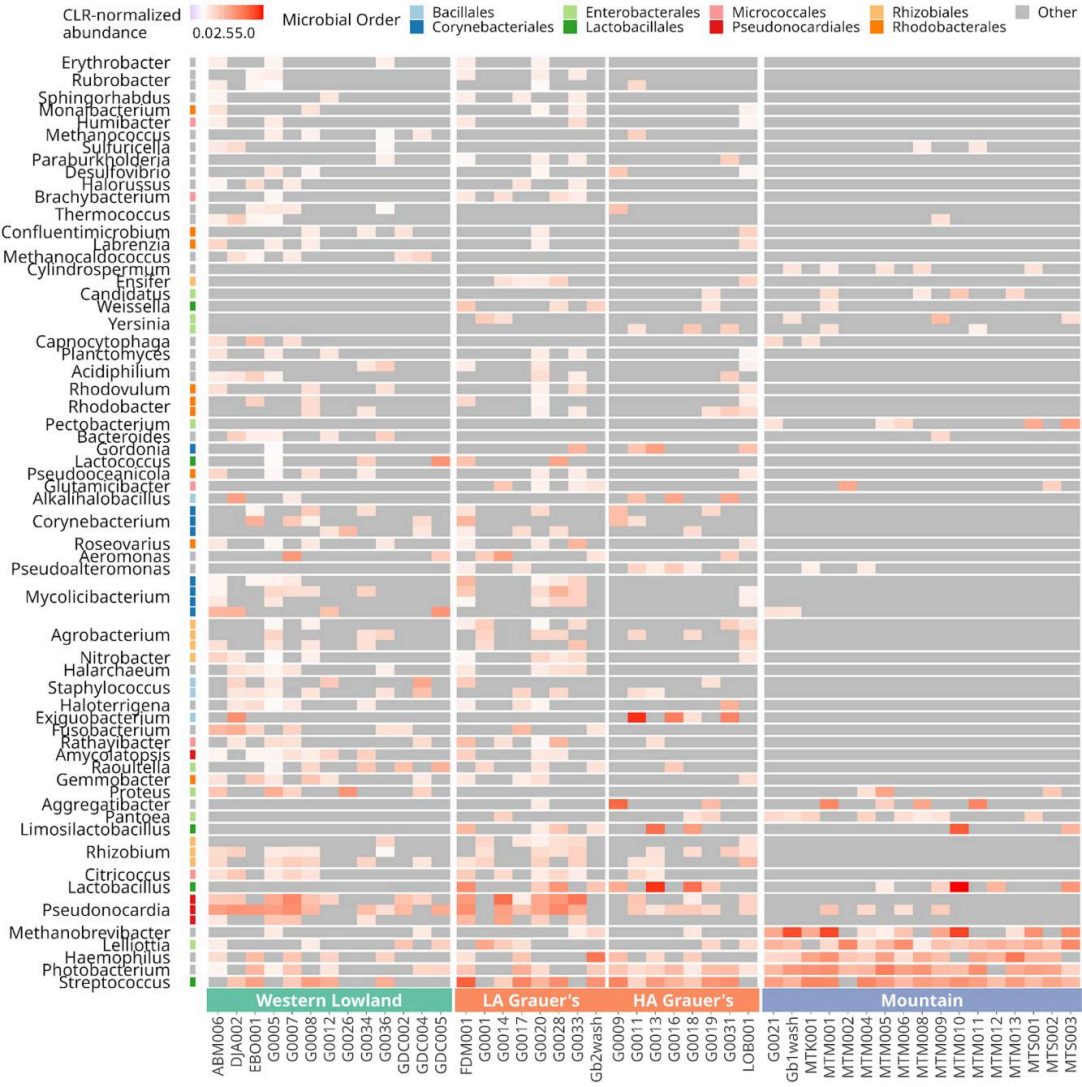
Heatmap depicting centered log-ratio (CLR) normalized abundances of 78 differentially abundant microbial taxa, marked by taxonomic order (y-axis), per sample (x-axis). Due to the CLR normalization, very low abundances or those equal to zero appear as negative values. For clarity, absent taxa (nonnormalized abundance equal to zero) are shown in gray. LA Grauer's, low-altitude Grauer's gorilla populations from ≤1000 masl; HA Grauer's, high-altitude Grauer's gorilla populations from >1000 masl.

Mountain gorillas, in particular, appeared to be missing many of these differentially abundant microbial taxa (fig. 4)—which is consistent with the observation of a somewhat lower microbial richness in this subspecies (fig. 2)—even after accounting for the effect of read depth. Microbial taxa that were absent in mountain gorillas belonged to the orders *Rhodobacteriales*, *Pseudonocardiales*, *Corynebacteriales* (represented mainly by *Corynebacterium* and *Mycolicibacterium*), *Bacillales* (including *Staphylococcus*), and *Rhizobiales* (including bacteria associated with the *Fabaceae* rhizosphere, such as *Agrobacterium deltaense, A. fabacearum* (Yan et al. 2017; Delamuta et al. 2020), and three *Rhizobium* species (Poole et al. 2018)). The presence of rhizosphere- and phyllosphere-associated taxa in western lowland and Grauer's gorillas may reflect habitat or dietary differences among the subspecies. Microbial taxa enriched in mountain gorillas primarily belonged to the orders *Enterobacterales* and certain *Lactobacillales*, like *Streptococcus* sp. and *Lactobacillus gasseri*. The microbiomes of Grauer's gorillas resembled those of western lowland gorillas, both overall (fig. 3 and table 1) and in terms of differentially abundant taxa (fig. 4). However, they also shared some similarities with mountain gorillas (e.g., a presence of *Limosilactobacillus*/*Lactobacillus* species, which were absent in western lowland gorillas), showing an intermediate or mixed composition (fig. 4).

### Gorilla Subspecies Harbor Functionally Distinct Oral Communities

After removing reads mapping to identified contaminants, the retained metagenomic reads were functionally characterized using HUMAnN2 (Franzosa et al. 2018) (Methods). The normalized abundances of gene families were regrouped under gene ontology (GO) terms, and were aggregated across microbial taxa. Functional results agreed with community-level taxonomic analyses, showing that functional profiles of mountain gorilla microbiomes differed significantly from the other two subspecies, whereas sequencing depth and dataset did not have a significant effect (table 2).

**Table 2.** Effects of Dataset and Host Subspecies on Microbiome Functional Profiles.

Terms	Within-factor Comparison	*R* ^2^	*P*
Read count		0.008	0.712
Dataset		0.030	0.220
Subspecies		0.100	0.060
	Western lowland versus Grauer's	0.013	0.695
	Western lowland versus mountain	0.124	**0.031**
	Grauer's versus mountain	0.132	**0**.**031**
Residuals		0.861	

PERMANOVA results, showing the effect size (*R*^2^) and *P* value of factors putatively contributing to the variance in abundance of biological processes among sampled microbiota. Pairwise PERMANOVA between subspecies shows the effect size (*R*^2^) and *P* values adjusted for false discovery rate. *P* values below 0.05 are shown in bold.

We identified differentially abundant biological processes, requiring that they were represented in at least 30% of the samples in at least one subspecies (Methods). Mountain gorillas were again distinct from the other subspecies, as 236 of the 262 differentially abundant processes were completely absent in this subspecies (supplementary table S5, Supplementary Material online). In contrast, western lowland and Grauer's gorillas were missing only 13 and 6 processes, respectively. Among six processes that were found in all three gorilla subspecies, mountain gorillas differed significantly from the others in four processes.

### Metagenome-assembled Genomes

We applied an iterative assembly and binning approach (Methods) to resolve initial MAGs using decontaminated sequencing reads from all samples and museum controls. We constructed a total of eight high-quality (>90% completion, < 5% contamination) and 27 medium quality MAGs (>50% completion, < 10% contamination), belonging to 24 distinct bacterial families (supplementary table S6, Supplementary Material online). Among these 35 MAGs, 25 could be classified to the genus level, with two MAGs assigned to provisional/uncultured genera (UBA8133 and RUG013; supplementary table S6, Supplementary Material online).

Fourteen MAGs reconstructed from dental calculus samples were also present in museum controls. Eight MAGs were present only in the skin sample and only at low abundances (supplementary fig. S4, Supplementary Material online). However, five MAGs were found at higher abundance in at least one of the museum controls compared with any gorilla dental calculus sample (supplementary fig. S4, Supplementary Material online). This includes MAGs of *Erysipelothrix*, two different *Planococcaceae*, as well as *Propionibacterium* and *Exiguobacterium*. These MAGs belong to genera that are common contaminants in metagenomic studies (i.e., *Propionibacterium*, which is a major genus of common skin bacteria (Salter et al. 2014)) and persistent in environmental reservoirs (i.e., *Erysipelothrix* (*Eriksson et al. 2014*)). Therefore, they were excluded from downstream analyses. To further authenticate our reconstructed MAGs, we used a collection of isolation source records from public databases of bacterial ecological metadata (supplementary table S7, Supplementary Material online). Taxa with a high proportion (>=25%) of isolation records from sources categorized as environmental/contaminant were considered as potential contaminants (supplementary table S6, Supplementary Material online). Using this approach, we identified 11 additional MAGs that represent likely contaminants (supplementary figs. S4 and S5, Supplementary Material online).

The remaining 19 MAGs recovered here are members of the oral microbiome and are present in databases of common oral taxa (Chen et al. 2010; Fellows Yates et al. 2021). This includes taxa commonly associated with dental plaque communities: *Rothia* (Tsuzukibashi et al. 2017), *Olsenella* (Socransky et al. 1998; Abusleme et al. 2013), *Corynebacterium* (Mark Welch et al. 2016), *Lautropia* (Gerner-Smidt et al. 1994), *Neisseria* (Donati et al. 2016), and *Actinomyces* (Kolenbrander et al. 2010). *Rothia* species are particularly abundant in gorillas compared with humans and other nonhuman primates (Fellows Yates et al. 2021). Three other MAGs were present in at least 65% of all samples: a MAG most closely related to *Neisseria* (present in 44 of the 46 samples), and the MAGs characterized to the family *Actinomycetaceae* and the genus *Lautropia*, both of which were found in all samples of mountain gorillas (supplementary fig. S4, Supplementary Material online).

**Fig. 5.**
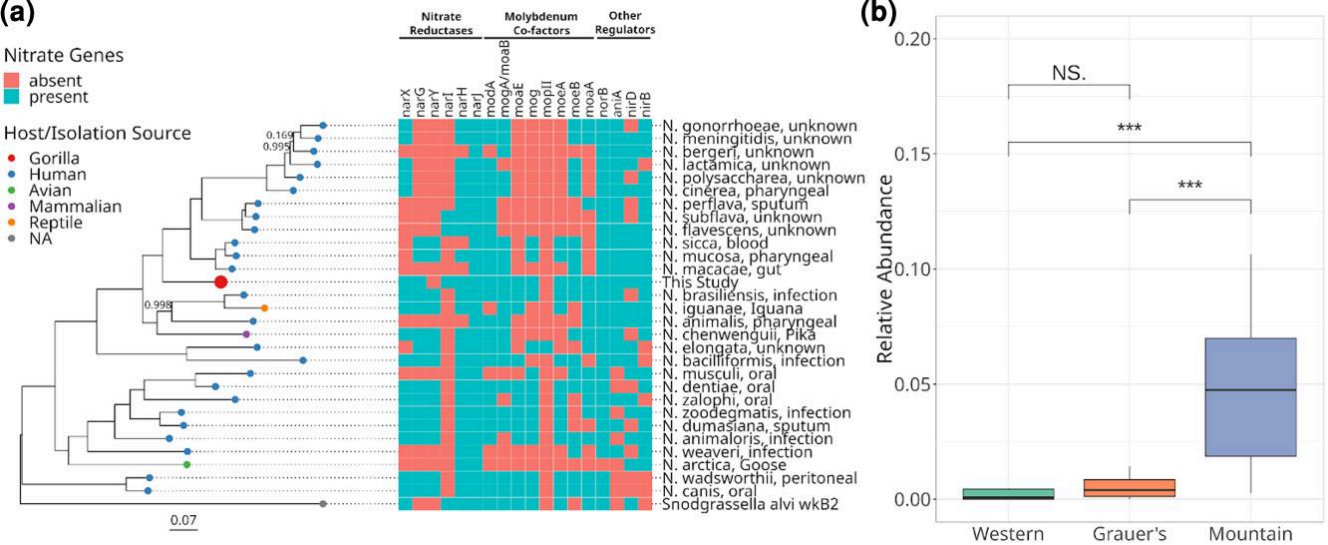
(*a*) Maximum-likelihood phylogeny based on the alignment of core gene sequences from the *Neisseria* MAG recovered from gorilla dental calculus and additional published genomes of the same genus. Tip points are colored by host species identity, and tip labels include the isolation source for each published genome. Scale bar units are the number of substitutions per site and node values represent the proportion of node support out of 100 bootstrap replicates, with unlabeled nodes having complete support (1.0). To the right, the presence of key genes involved in the reduction of nitrate in the oral cavity is shown for each *Neisseria* species. (*b*) Relative abundances (CLR transformed) of the *Neisseria* MAG in different gorilla subspecies. The results of a Wilcoxon test are denoted by brackets above boxplots (NS, not significant; *** = *P* > 0.001).

For two MAGs of high completeness and high prevalence (*Neisseria* and *Limosilactobacillus gorillae*), we produced an alignment of core genes to investigate how they relate to the known diversity of these taxa. The *Neisseria* MAG clustered with a subset of *Neisseria* species isolated exclusively from humans and was more divergent from *Neisseria* species that formed a clade with isolates from other animals (fig. 5*a*). Its phylogenetic placement suggests a distinct and undescribed *Neisseria* taxon. *Limosilactobacillus gorillae* (supplementary fig. S6, Supplementary Material online) is a species previously isolated from the feces of mountain and western lowland gorillas (Tsuchida et al. 2015, 2018). The *L. gorillae* MAG recovered from dental calculus grouped as a sister taxon to the gorilla fecal isolate, but was distinct from fecal isolates of other primates (supplementary fig. S6*a*, Supplementary Material online).

Oral bacteria play an important role in the metabolism of inorganic nitrate and its reduction to nitrite and nitric oxide (NO), which is essential for the regulation of cardiovascular, metabolic, and neurological processes (Hezel and Weitzberg 2015; Bryan et al. 2017). Several oral nitrate-reducing bacteria, including *Rothia*, *Neisseria*, *Veillonella*, *Corynebacterium*, *Actinomyces*, *Selenomonas*, *Propionibacterium*, *Fusobacterium*, and *Eikenella (Rosier*et al*2022)*, were present in gorilla dental calculus samples (supplementary fig. S4, supplementary table S4, Supplementary Material online). We used MAGs of *Rothia*, *Neisseria*, and *Veillonella*, which were prevalent in the study samples (supplementary fig. S4, Supplementary Material online) to analyze their repertoire of nitrate-reducing genes. For *Neisseria*, we could confirm the presence of five nitrate reductases (*nar*), seven genes that provide the cofactor molybdenum to the nitrate reductase enzymes, and four additional genes involved in the regulation of nitrate and nitrite reduction (*aniA*, *nirB*, *nirD*, *norB*; fig. 5*a*). Only two genes involved in nitrate reduction, *narY* and *mopII*, were not found in the gorilla dental calculus MAG. These genes are also absent from other nitrate-reducing oral bacteria (Rosier et al. 2020) and appear nonessential. Genomic data thus suggests that *Neisseria* isolated from gorilla dental calculus may have the full capacity to reduce nitrate. The *Neisseria* MAG recovered in this study was significantly more abundant in mountain gorillas compared with the other host subspecies (fig. 5*b*).

*Rothia* and *Veillonella* possessed a smaller repertoire of nitrate-reducing genes compared with *Neisseria*. The *Rothia* MAG recovered in this study had several nitrate reductases and molybdenum cofactor genes, but they differed from genes present in *R. dentocariosa*, a bacterium well studied for its nitrate-reducing capacity (supplementary fig. S7, Supplementary Material online; *Rosier*et al*. 2020)*. We were also able to identify nine genes involved in nitrate metabolism in *Veillonella*, another taxon known to reduce nitrate in the oral cavity (Hyde, Andrade, et al. 2014). Similar to *Neisseria*, *Veillonella* was significantly more abundant in mountain gorillas than in the other two gorilla subspecies, whereas the abundance of *Rothia* did not differ among gorilla subspecies (supplementary fig. S7*b*and S7*d*, Supplementary Material).

### Altitude may Drive Oral Microbiome Composition

Both taxonomic and functional analyses suggest that Grauer's gorilla oral microbiomes are more similar to western lowland gorillas than to mountain gorillas, despite sharing a close evolutionary relationship and an adjacent geographic range with the latter. However, the distribution ranges of all three gorilla subspecies differ in elevation, with mountain gorillas occurring at the highest altitudes. As altitude can influence temperature, humidity, and food diversity, which in turn can influence microbial communities, we performed partial Mantel tests between altitudinal distances and taxonomic (Jaccard/Aitchison distances) or functional (Euclidean distance) composition of the oral microbiome, while accounting for log-transformed geographical distance. Geographic location and altitude of the specimens were approximated based on museum records. However, when considering the entire dataset, we did not detect a correlation between altitude and either taxonomy or function (Mantel test: R ranging from −0.13 to 0.06, *P* > 0.12).

Since Grauer's gorillas occupy the widest altitudinal range of all gorilla subspecies, we divided Grauer's gorilla samples into high- (>1000 masl, *n* = 8) and low-altitude (≤1000 masl, *n* = 8) groups and tested for differentiation of the oral microbiome both between these groups and among host subspecies. We found that the oral microbiome composition of western lowland and low-altitude Grauer's gorillas was indistinguishable, whereas mountain gorillas differed significantly from all subspecies, independent of altitude grouping (supplementary table S8, Supplementary Material online). This result can be explained by the altitudinal distribution of our samples, with mountain gorilla samples originating from an area that is at least 1000 meters higher than all other samples (supplementary fig. S8, Supplementary Material online).

Despite these differences, high-altitude Grauer's gorillas showed considerable overlap with mountain gorillas in the first two axes of a PCoA based on Aitchison distances (supplementary fig. S9, Supplementary Material online), which prompted us to further investigate the effect of altitude on the oral microbiome. To this end, we identified a set of differentially abundant taxa between mountain and western lowland gorillas (*n* = 41) and compared their abundance in Grauer's gorillas from different altitudes. We observed no significant difference between low-altitude Grauer's and western lowland gorillas and between high-altitude Grauer's and mountain gorillas when considering these differentially abundant taxa, whereas all other comparisons were significant (*P* ≤ 0.006, table 3). This result supports the notion that altitude has an effect on the oral microbiome composition.

**Table 3.** PERMANOVA Results of Microbial Taxa Identified as Differentially Abundant Between Mountain and Western Lowland Gorillas.

Comparison	Jaccard		Aitchison	
	*R* ^2^	*P*	*R* ^2^	*P*
Mountain versus low-altitude Grauer's	0.220	**0.002**	0.222	**0**.**003**
Mountain versus high-altitude Grauer's	0.095	0.052	0.078	0.159
Western lowland versus low-altitude Grauer's	0.077	0.335	0.059	0.536
Western lowland versus high-altitude Grauer's	0.211	**0**.**003**	0.283	**0**.**004**
Low-altitude Grauer's versus high-altitude Grauer's	0.243	**0**.**002**	0.246	**0**.**006**

We report the PERMANOVA results using both Jaccard (reflecting presence–absence of taxa) and Aitchison (reflecting relative abundance of taxa) distances. Corrected *P* values below 0.05 are shown in bold.

### Dental Calculus Reflects Dietary Differences of Gorilla Subspecies

Dietary characterization from dental calculus is notoriously difficult (Mann et al. 2020). Yet, our results suggest that ecological factors play a role in gorilla oral microbiome composition and diet is well-known to impact the oral microbiome and differs considerably among gorilla taxa. Hence, we investigated the dietary signature preserved in gorilla dental calculus samples. After removing reads assigned to bacteria and viruses, we performed taxonomic classification of the eukaryotic diversity using Kraken2 with the NCBI “nt” database. Despite applying extensive decontamination procedures (Methods) and removing taxa with few supporting reads (<10), we still recovered erroneous assignments, such as mollusks or other mammals that are common false-positives in dietary analyses (Mann et al. 2020) and are unlikely to be consumed by gorillas. Therefore, we restricted our analyses to eukaryotic families that are established components of the gorilla diet (Remis et al. 2001; Rogers et al. 2004; Yamagiwa et al. 2005; Rothman et al. 2014; Michel et al. 2022). The majority of the 314 genera (65 families) detected in our dataset (supplementary fig. S10, Supplementary Material online) were plants (*n* = 300), with some insects, specifically ants (*n* = 9 genera), and lichen-forming fungi (family *Parmeliaceae*, *n* = 5 genera). Due to the low number of eukaryotic reads (191 reads on average, per sample and taxon with >10 reads), we were unable to systematically analyze DNA damage profiles (Mann et al. 2020). However, the damage profiles for the plant genus *Galium* (supplementary fig. S11*a*, Supplementary Material online), which was highly abundant in our samples, showed nucleotide misincorporation rates typical for samples of around 100 years of age (Sawyer et al. 2012), suggesting its authenticity (supplementary fig. S11*b*, Supplementary Material online).

**Fig. 6.**
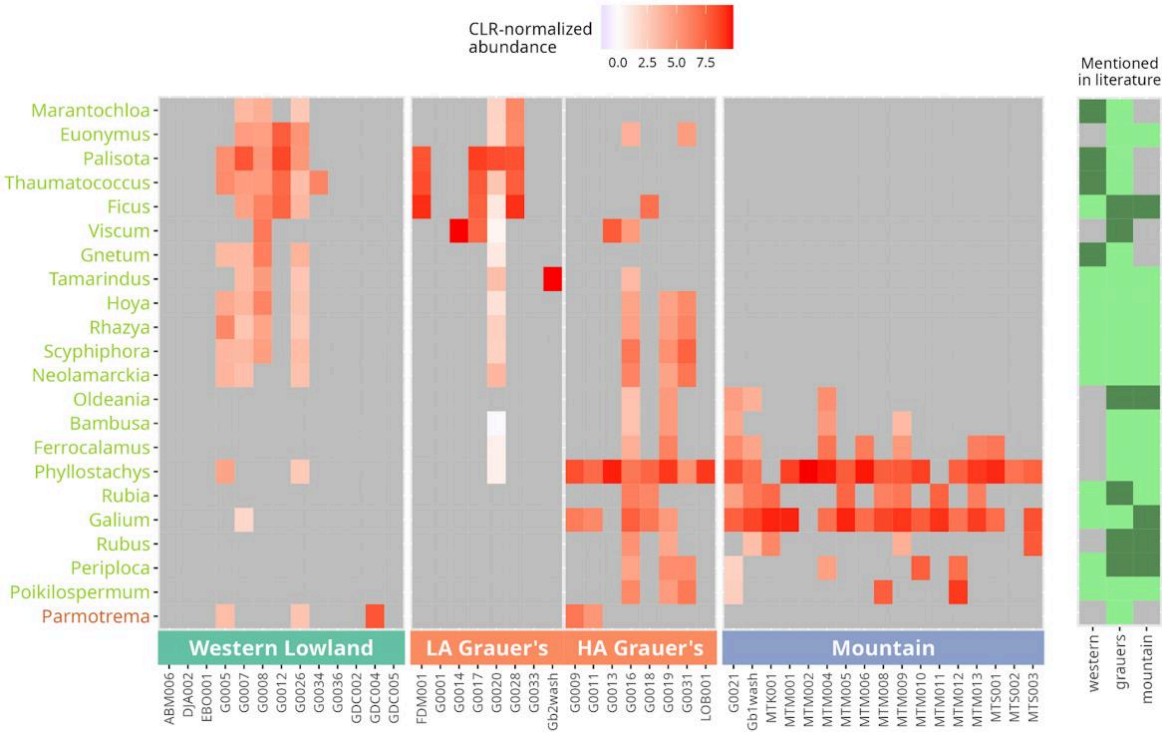
Heatmap based on normalized abundances for differentially abundant dietary taxa at the genus level. For clarity, taxa with an abundance equal to 0 are shown in gray. The bars on the right indicate if the family (light color) or the specific genus (dark color) has been reported as part of the diet of each gorilla subspecies (gray: no mention). Genus names are shown on the left, with plants in green (light) and fungi in brown (dark). LA Grauer's, low-altitude Grauer's gorilla populations from ≤1000 masl; HA Grauer's, high-altitude Grauer's gorilla populations from >1000 masl.

We detected 22 genera that differed in abundance across gorilla subspecies, all belonging to plants, except for the lichen genus *Parmotrema* (fig. 6; supplementary table S9, Supplementary Material online). Although it is possible that this lichen is unintentionally ingested by gorillas (e.g., during nest construction), it could also be a misclassification of the cosmopolitan genus *Usnea*, which is an occasional part of Grauer's gorilla's diet (Yamagiwa et al. 2005). The broad dietary patterns observed here agree with the literature reports. For instance, bamboo—represented here by the African genus *Oldeania*, as well as Asian genera *Phyllostachys*, *Bambusa,* and *Ferrocalamus*, which are likely misclassifications of African bamboo species (Brealey et al. 2020)—was mainly detected in mountain and Grauer's gorillas, as expected (Yamagiwa et al. 2005; Rothman et al. 2014). *Marantochloa* and *Thaumatococcus* of the *Marantaceae* family were detected in western lowland and Grauer's gorillas, which are known to consume them (Rogers et al. 2004; Michel et al. 2022). The family *Fabaceae* is consumed by all gorilla subspecies, but the specific genus *Tamarindus* is likely a misidentification of another member of this family, as *Tamarindus* itself has not been reported to be part of the gorilla diet. Grauer's gorillas appear to have the most diverse diet of the three subspecies, consuming foodstuffs typical of both western lowland and mountain gorillas (fig. 6).

Having characterized qualitative dietary profiles of gorilla subspecies, we investigated if the dietary composition is affected by altitudinal differences in our dataset. We find strong support for an effect of altitude on gorilla diet, uncovering significant differences between low- and high-altitude Grauer's gorillas (*P* < 0.05), but no differences between low-altitude Grauer's and western lowland gorilla or high-altitude Grauer's and mountain gorillas (considering at least one of the distance metrics, supplementary table S10, Supplementary Material online). Furthermore, we detected a significant, albeit weak positive relationship between microbial and dietary distances in our data (*R*^2^ = 0.051, *P* = 0.048; supplementary fig. S12, Supplementary Material online).

## Discussion

Using three gorilla subspecies, we investigated how evolutionary relationships and ecological factors shape the dental calculus oral microbiome in closely related host taxa. Following our predictions for the role of ecology in structuring the oral microbiome communities and against the dominant influence of host phylogenetic relationships, we observe strong differences in taxonomic composition, relative abundance, and functional profiles of microbial taxa between closely related and geographically proximate Grauer's and mountain gorillas (tables 1 and 2, supplementary table S4, Supplementary Material online). Conversely, we find more similarities between Grauer's and western lowland gorillas that are more distantly related and separated by the Congo Basin. When considering differentially abundant microbial taxa and dietary profiles, Grauer's gorillas appear intermediate, displaying a combination of taxa found in both western lowland and mountain gorillas (figs. 4 and 6). One of the strongest differences between the three gorilla subspecies is the altitude of their ranges, which in turn influences diet. We show that altitudinal differences, likely primarily through their effect on diet, but possibly also by imposing additional selective pressures on the host (see below), impact the oral microbiome of gorilla subspecies and populations (figs. 4 and 6, supplementary figs. S9, S10, and S12, Supplementary Material online; tables 1–3, supplementary tables S8 and S10, Supplementary Material online).

Our findings deviate from previous studies that identified a phylogenetic signal in the oral microbiome in wild animals. However, these studies compared distantly related species like chimpanzees, humans (Li et al. 2013), other great apes (Boehlke et al. 2020), and even more diverse groups of mammals (Brealey et al. 2020; Ozga and Ottoni 2021). Often, the effects of host phylogenetic relationships and ecology could not be decoupled. For instance, Smith et al. (2021) found differences among three snake species that each occupied a distinct ecological niche. Similarly, Soares-Castro et al. (2019) studied three marine mammals, among which the most evolutionary divergent species was also ecologically distinct, resulting in the effects of ecology being indistinguishable from those of evolutionary relationships. Our results align well with other studies investigating multi-species communities in uncovering the importance of ecological conditions and dietary differences (Poulin 1995; Li et al. 2013; Wade 2013; Beer et al. 2019; Kartzinel et al. 2019; Youngblut et al. 2019; Janiak et al. 2021).

Yet, ecological effects on a short evolutionary scale, as observed in this study, do not preclude codiversification of the host and its associated microbiome over a longer evolutionary time. They rather point to the presence of rapid and dynamic responses of the host-associated microbial communities to environmental conditions, potentially through the seeding of the dental calculus oral microbiome, at least to some extent, with environmental taxa (see below).

### Oral Microbiome Diversity Might Reflect Ecology or Diet

Our analyses indicate that ecological differences, approximated here by altitude, may be the primary factor shaping oral microbiome diversity. Specifically, when considering taxa that are differentially abundant between western lowland and mountain gorillas (the two altitudinal extremes, supplementary fig. S8, Supplementary Material online), low-altitude Grauer's gorilla populations are indistinguishable from western lowland gorillas, whereas high-altitude Grauer's gorilla populations show no differences to mountain gorillas (table 3). We note that all mountain gorillas in our dataset were collected above 3000 masl, notably higher than any of Grauer's gorilla samples (600–2100 masl; supplementary table S1, Supplementary Material online, supplementary fig. S8, Supplementary Material online). Hence, it is not surprising that the main differences we detect in the global analyses of microbial composition are between mountain gorillas and all other subspecies (supplementary table S8, Supplementary Material online).

Because altitude strongly influences gorilla diet (Doran et al. 2002; Ganas et al. 2004; Rogers et al. 2004; van der Hoek et al. 2021; Michel et al. 2022) and diet, in turn, affects oral microbiome composition (Wade 2013; Janiak et al. 2021), it is possible that the observed differences can be primarily explained by dietary differences among the gorilla subspecies. Hence, we characterized dietary profiles from dental calculus samples. Several previous studies have identified important caveats associated with dietary analyses from this source material (Ottoni et al. 2019; Mann et al. 2020; Haas et al. 2022). Sample contamination, a low proportion of eukaryotic reads, and the sparsity of reference databases for eukaryotic taxa can lead to analytical biases. In addition, reference genome contamination with microbial genes can result in false positive taxonomic assignments (Mann et al. 2020). In the present study, the authenticity of all putative dietary taxa could not be systematically evaluated using DNA postmortem damage patterns due to the low abundance of eukaryotic reads. However, we detect typical damage patterns in a highly abundant dietary taxon (*Galium*; supplementary fig. S11, Supplementary Material online), suggesting the authenticity of our dietary results. Despite these limitations, we detect subspecies-specific differences in dietary profiles as recovered from dental calculus, which are broadly in agreement with known gorilla diets (fig. 6; Remis et al. 2001; Rogers et al. 2004; Yamagiwa et al. 2005; Rothman et al. 2014; Michel et al. 2022). Our dietary analyses recapitulate the patterns observed in microbial profiles. Specifically, we find that dietary profiles differ significantly between mountain and western lowland gorillas but not with high-altitude Grauer's gorillas (supplementary table S10, Supplementary Material online). We also observe a weak but significant correlation between dietary and microbial profiles obtained from dental calculus samples (supplementary fig. S12, Supplementary Material online), which further supports the link between diet and oral microbiome composition. Dental calculus of Grauer's gorillas appears to contain dietary items present in both western lowland and mountain gorillas (fig. 6 and supplementary fig. S10, Supplementary Material online). This is in line with previous studies (Remis et al. 2001; Rogers et al. 2004; Yamagiwa et al. 2005; Rothman et al. 2014; Michel et al. 2022), which report greater dietary richness and considerable overlap in the diet of Grauer's gorillas with western lowland (30 of the 41 plant families) and mountain (30 of the 45 plant families) gorillas. In contrast, only 16 dietary families are consumed by both western lowland and mountain gorillas.

We found no effects of sex on the dietary or oral microbiome composition, when considering either museum records or molecular sex assignments (supplementary table S3, Supplementary Material online). This is in agreement with studies in western lowland, Grauer's and mountain gorillas that detected no sex-specific differences in diet using different approaches (Doran et al. 2002; Rogers et al. 2004; Harty et al. 2022; Michel et al. 2022) and only a weak effect of sex on the gut microbiome (Michel et al. 2022). Similarly, we found no effect of individual age on either dietary or microbial composition (supplementary table S3, Supplementary Material online); however, only two age categories could be considered (adults and juveniles + subadults). Infants are rarely present in museum collections and have no or insufficient dental calculus deposit for sampling.

It is intriguing to speculate that the local environment itself could affect the composition of the dental calculus community by serving as a source of at least some colonizing taxa, for example via the consumed phyllosphere and rhizosphere. A recent study of the human oral microbiome found that specific taxa within the dental plaque community are evolutionarily close to environmental bacteria, whereas those living on the tongue surface are more closely related to other host-associated taxa (Shaiber et al. 2020). It is hence possible that dental plaque microbial communities are more affected by the environment than other host-associated microbiomes. In gorillas, we detected microbial taxa that likely have a dietary origin and differ in abundance among host subspecies, possibly reflecting dietary differences. In particular, bacteria associated with the *Fabaceae* rhizosphere, including *Agrobacterium deltaense*, *A. fabacearum* (Yan et al. 2017; Delamuta et al. 2020), and three *Rhizobium* species (Poole et al. 2018) may be derived from the consumed dietary items or accidentally ingested soil. These bacteria were significantly more abundant in the dental calculus of western lowland and Grauer's gorillas than in mountain gorillas (fig. 4; supplementary table S4, Supplementary Material online). Two lines of evidence provide a link between these microbial taxa and dietary differences among the gorilla subspecies. First, our data suggest a higher abundance of the family *Fabaceae* in western lowland and Grauer's gorillas (supplementary fig. S10, Supplementary Material online) compared with mountain gorillas. Second, although no comparative data exist on the prevalence of roots and rhizomes in the diet of different gorilla species, indirect evidence suggests that western lowland gorillas consume more roots than mountain gorillas. Behavioral observations report frequent root and rhizome consumption in western lowland gorillas of all ages (Fletcher and Nowell 2008) and comparative dentition analyses show increased tooth wear in western lowland gorillas compared with mountain gorillas, in which rates of tooth wear are well explained by the time spent feeding on roots (Galbany et al. 2016).

### Metagenome-assembled Genomes add to the Understanding of Wild Microbiomes

The recovery of high-quality MAGs from novel environments, such as the dental calculus of nonhuman animals, is an important complementary method that may uncover unknown microbial lineages (Jiao et al. 2021). Several high- and medium-quality MAGs constructed in this study were highly abundant in the dental calculus samples and belong to taxa that are members of the oral microbiome (supplementary figs. S4 and S5, Supplementary Material online). Many of these MAGs were distinct from the evolutionary closest reference genomes (fig. 5, supplementary figs. S6 and S7, Supplementary Material online), suggesting that they may represent undescribed bacterial lineages.

We recovered a near-complete MAG of *Limosilactobacillus gorillae*, which was more closely related to a fecal isolate from a captive gorilla than to a fecal isolate from another primate (supplementary fig. S6*a*, Supplementary Material online). Usually associated with gorilla feces (Tsuchida et al. 2014), the presence of *L. gorillae* within the oral microbiome may be the result of coprophagy, a common behavior among wild gorillas (Graczyk and Cranfield 2003). However, questions remain regarding the persistence of fecal-associated bacteria within dental calculus and their potential function within the plaque biofilm. Coprophagic behavior is suggested to serve as a route for vertical or horizontal transmission of gastrointestinal microbiota between individuals (Abusleme et al. 2020) and may have a stabilizing impact on host-associated microbial communities overall (Bo et al. 2020).

We also reconstructed MAGs of *Neisseria*, *Rothia*, and *Veillonella* and confirmed the presence of nitrate-reducing genes in these members of the gorilla oral microbiome (figs. 5*a*, supplementary fig. S7, Supplementary Material online). The reduction of nitrate to nitrite from dietary sources by oral bacteria provides the precursor to nitric oxide (NO), an important signaling and effector molecule (Jones et al. 2021). Along with multiple benefits to circulatory and cardiovascular health (Lundberg et al. 2018), NO is increased in response to hypoxic stress (Levett et al. 2011; Feelisch 2018), and results in increased blood oxygen levels (Beall et al. 2012). Human populations adapted to high-altitude exhale higher concentrations of NO compared with lowland populations (Beall et al. 2001; Erzurum et al. 2007). For these reasons, regulation of NO metabolism is thought to be beneficial to high-altitude adaptation (Beall et al. 2012). Mountain gorillas live at an elevation of up to 3,800 masl (Williamson et al. 2013) and, hence, experience hypoxic stress (Ruff et al. 2022). They also show a higher abundance of *Neisseria* and *Veillonella* compared with the other two gorilla subspecies (figs. 5*b* & supplementary fig. S7*d*, Supplementary Material online), which live at considerably lower altitudes (supplementary fig. S8, Supplementary Material online). Although it is not known if dietary nitrate amounts differ among gorilla subspecies, the primarily herbivorous diet of mountain gorillas is naturally rich in nitrate. A high abundance of nitrate-reducing oral taxa in mountain gorillas could offer physiological benefits for their high-altitude lifestyle. Provided the nitrate-reducing activity of these bacteria can be demonstrated experimentally, our finding is particularly intriguing, as comparative genomic analyses have failed to uncover host-encoded genes related to high-altitude adaptation in mountain gorillas (Xue et al. 2015). Our results may suggest an important role of the oral microbiome in promoting host adaptations to high-altitude environments, as has been shown in the gut (Zhang et al. 2016).

## Conclusions

Our study is, to our knowledge, the first to investigate the evolution of the dental calculus oral microbiome at the early stages of the speciation process of the host and adds to the new but growing field of research on the microbiomes of wild animals. We find that in closely related species, evolutionary relationships are less important than ecology in explaining the taxonomic composition and function of the oral microbiome. Host-associated microbial communities have been proposed to contribute to host adaptation, partly because they can respond rapidly to changing environmental conditions (Alberdi et al. 2016). We find that taxa enriched in the mountain gorilla oral microbiome may facilitate their high-altitude lifestyle through increased nitrate reduction potential and the associated physiological benefits. Our discovery of distinct phylo- and rhizosphere taxa suggests a close connection between this host-associated microbiome and the local environment. Colonization of the host by environmental taxa with beneficial functions in local adaptation and health may present an exciting evolutionary route to be explored in the future.

## Materials and Methods

### Sample Collection

The study dataset consisted of dental calculus samples from 57 specimens of wild gorillas belonging to three subspecies: 16 western lowlands gorillas, 22 Grauer's gorillas, and 19 mountain gorillas (supplementary table S1, Supplementary Material online). Dental calculus samples of western lowland gorillas were collected at the Royal Museum for Central Africa (RMCA, Tervuren, Belgium) and the Cleveland Museum of Natural History (CMNH, USA). Samples of mountain gorillas came from the Swedish Museum of Natural History (Naturhistoriska Riksmuseet—NRM, Stockholm, Sweden) and the RMCA. Samples of Grauer's gorillas were collected primarily at RMCA, with a few samples from NRM and the Royal Belgian Institute of Natural Sciences (RBINS, Brussels, Belgium). Our dataset consisted of newly generated shotgun data from 26 specimens and published gorilla dental calculus sequences: two samples from Brealey et al. (2020) and 29 samples from Fellows Yates et al. (2021). For simplicity, the two samples from Brealey et al. are considered as part of the “newly generated” dataset, as they were processed in the same facilities, with the same protocol and sequenced in the same manner as the 26 new samples. In contrast, the samples from Fellows Yates et al. (2021) were processed and sequenced using slightly different methods (see next section and supplementary table S1, Supplementary Material online) and are referred to as the “Fellows Yates et al. 2021” dataset. We also included sequences from 34 published and newly generated extraction blanks, 11 library preparation blanks, and four museum controls (supplementary table S1, Supplementary Material online). The museum controls were swabs of a specimen shelf and the surface of a brown bear (*Ursus arctos*) skull, both from NRM. We also included data from two previously published gorilla specimens (van der Valk et al. 2017; van der Valk et al. 2019): A petrous bone sample from a specimen at NRM and a skin sample from RMCA (ENA accession numbers: ERR2503700 and ERR2868193, respectively), from which host reads were removed.

### Preparation of Genomic Libraries and Metagenomic Shotgun Sequencing

All samples were processed in cleanroom facilities following methods and routines for working with ancient DNA. DNA was extracted using the protocol by Dabney et al. (2013) with slight changes, as described in Brealey et al. (2020) and Fellows Yates et al. (2021). The datasets differed in library preparation protocol, indexing strategy, sequencing platform, read length, and sequencing depth (supplementary table S1, Supplementary Material online). Samples from four specimens were processed in both datasets and were used to assess putative batch effects (supplementary fig. S2, Supplementary Material online), retaining only the sample with the largest number of reads per pair for analyses.

For the newly generated data, we followed the protocol as detailed in Brealey et al. (2021). Briefly, dental calculus samples ranging in weight from < 5 to 20 mg were surface-decontaminated using UV light (10 min at 254 nm) and washed in 500 µl of 0.5 M ethylenediaminetetraacetate for 1 min (Ozga et al. 2016; Brealey et al. 2020, 2021). DNA was extracted using a silica-based method (Dabney et al. 2013) in batches of at most 16 samples with two negative controls. DNA was eluted in 45 µl of EB buffer (10 mM tris-hydrochloride, pH 8.0; QIAGEN, Netherlands) supplemented with 0.05% (v/v) Tween-20.

Double-stranded genomic libraries for all samples were prepared following the double indexing protocol (Meyer and Kircher 2010; Dabney and Meyer 2012). Samples of the newly generated dataset included double in-line barcodes to guard against index hopping (Brealey et al. 2020; van der Valk et al. 2020). Library blanks were included for each batch of approximately 20 samples. The appropriate number of indexing cycles was estimated using a quantitative PCR assay and ranged from 8 to 20. Individual libraries were purified with Qiagen MinElute columns. After selecting fragments of approximately 100–500 bp with AMPure XP beads (Beckman Coulter, IN, USA), libraries were pooled (1.5 µl of each library) and sequenced on two Illumina NovaSeq S2 flowcells using paired-end 100 bp read length and V1 sequencing chemistry.

### Preprocessing of Sequencing Data

We removed the poly-G tails with fastp (V0.20.0; Chen et al. 2018) and unpaired reads with BBTools `repair.sh` (V38.61b; Bushnell 2014). For newly generated data, we used AdapterRemoval (V2.2.2; Schubert et al. 2016) to clip adapters, trim reads of minimum phred quality (>=30) and length (30 bp), and merged forward and reverse reads. The unmerged reads were excluded from downstream analysis (supplementary table S1, Supplementary Material online). In-line barcodes in newly generated data were trimmed using a Python script (Brealey et al. 2020). Read quality filtering was performed using PrinSeq-Lite (V0.20.4; Schmieder and Edwards 2011) with a mean base quality threshold of 30. PCR duplicates were removed using a Python script (Brealey et al. 2020). All commands and scripts are available at 10.5281/zenodo.6861585.

We removed reads from the internal Illumina sequencing control *phi*X bacteriophage, the gorilla host, and the most likely contaminant, human, by mapping against the *phiX* (GenBank: GCA_000819615.1), western lowland gorilla (GCF_000151905.2), and human (GRCh38) reference genomes using BWA-MEM (V0.7.17; Li and Durbin 2009). Unmapped reads were retained using SAMtools (V1.12; Danecek et al. 2021) and converted to FASTQ for downstream analysis using BEDtools (V2.29.2; Quinlan and Hall 2010). Extraction blanks, library blanks, and museum controls were only mapped to the human genome. The unmapped reads were used in oral microbiome and dietary analyses. The mapped reads were used in host subspecies identification (supplementary tables S2 and S11, Supplementary Methods, Supplementary Material online). Molecular sex assignment was performed using decontaminated data prior to host removal (Supplementary Methods, Supplementary Material online).

### Initial Taxonomic Classification and Decontamination

#### Taxonomic Classification With Kraken2/Bracken

After removing host and human reads, the unmapped reads were taxonomically classified using Kraken2 (V2.1.1; Wood et al. 2019) with the standard database (which includes all bacterial, archaeal, and viral genomes from NCBI; accessed September 1, 2021) and default parameters. Abundances were re-estimated at the species level using Bracken (V2.6.2; Lu et al. 2017). The raw species table and the corresponding metadata were analyzed using phyloseq (V1.34.0; McMurdie and Holmes 2013) in R (V4.0.4; R Core Team 2015). Taxonomic information was retrieved using the “classification” function of the taxize package (V0.9.99; Chamberlain and Szöcs 2013).

#### Removal of Low-quality Samples and Contaminant Taxa

Samples containing less than 300,000 processed reads were excluded from downstream analysis (supplementary fig. S13, Supplementary Material online). We then used FEAST (V0.1.0; Shenhav et al. 2019) to evaluate the composition of microbial communities using user-provided reference microbiomes: human calculus (*n* = 5; Mann et al. 2018) and human plaque (*n* = 5; Human Microbiome Project Consortium 2012; Lloyd-Price et al. 2017) representing the oral microbiome, and human gut (*n* = 5; Human Microbiome Project Consortium 2012; Lloyd-Price et al. 2017), human skin (*n* = 5; Oh et al. 2014), tundra soil (*n* = 5; Johnston et al. 2016), and laboratory contaminants excluding human sequences (*n* = 4; Salter et al. 2014) representing contaminants (supplementary table S12, Supplementary Material online). Based on the distribution of oral proportions from FEAST (supplementary fig. S3*b*, Supplementary Material online), gorilla dental calculus samples with a cumulative oral proportion lower than 3% were excluded from further analyses.

For the retained samples, we then applied a multi-step approach to remove putative contaminant microbial taxa. First, we used the R package decontam (V1.10.0; Davis et al. 2018) which identifies contaminants based on increased prevalence in blanks and an inverse relationship between abundance and input DNA quantity in each sample. Decontamination was performed separately for “newly generated” and “Fellows Yates et al. 2021” datasets, with different thresholds for prevalence (0.2 and 0.3, respectively), chosen to minimize the exclusion of oral taxa (Chen et al. (2010) and Fellows Yates et al. (2021); supplementary fig. S14, Supplementary Material online). All taxa identified as contaminants were then removed from the full dataset, regardless of which subset they were identified in.

Second, we performed abundance filtering on the entire dataset, setting each taxon to 0 if it had relative abundance <0.005% in a given sample, which retained the highest number and proportion of oral taxa (supplementary table S13, Supplementary Material online). Third, a given taxon was removed if it had a higher relative abundance in any of the four museum controls than in any of the samples. Lastly, we directly removed genera that have been identified as common contaminants in typical molecular and specialized ancient DNA laboratories (Salter et al. 2014; Weyrich et al. 2019) and are not present in oral databases (HOMD; accessed July 14, 2021; Chen et al. 2010; Fellows Yates et al. 2021). The authenticity of taxa that were present in both contaminant lists and the oral databases, and had a sufficient number of reads, was investigated using mapDamage2 (V2.0.9; Jónsson et al. 2013). To this end, we identified the sample with the highest abundance for each taxon (minimum 10,000 reads classified in Kraken2) and mapped the reads to the respective reference genome. We assessed the presence of postmortem DNA damage as deamination (C-to-T in the 3′ end and G-to-A in the 5′ end) frequency above 0.02 in at least two of the three terminal positions. This rule was employed because in-line barcodes can create atypical DNA damage patterns in metagenomic data, showing lower damage at the 1st terminal position (Brealey et al. 2020). Bacterial taxa with no damage were assumed to be modern contaminants and were, therefore, removed. Taxa with too few reads to be tested were automatically retained. The resulting dataset was used for downstream taxonomic analyses.

#### Sequence-level Decontamination

Prior to the functional characterization of microbial communities and MAGs reconstruction, we removed sequencing reads from taxa identified as contaminants in the above analyses (*n* = 167) using KrakenTools (V1.2; Wood et al. 2019). This approach retained sequencing reads of all taxa not identified as contaminants (i.e., those included in the final taxonomic dataset), but also a large number of unclassified reads which could belong to contaminant taxa. To filter these reads, we constructed a reference dataset containing the genomes of the identified contaminants (*n* = 167) and a selection of abundant noncontaminant taxa from our dataset (taxa with more than 10,000 reads in at least one sample, *n* = 72). The noncontaminant taxa were included to avoid forced mapping to the contaminants. We obtained one genome per taxon from NCBI assemblies (NCBI ftp accessed April 14, 2021, available at 10.5281/zenodo.6861585) for most of our taxa. Reference genomes were not available for 22 contaminant and 11 noncontaminant taxa, so we used a different genome of the same genus. We then mapped reads from the previous decontamination step to this combined reference dataset (consisting of 155 contaminant genomes and 61 noncontaminant genomes) and retained unmapped reads and reads that mapped primarily to the noncontaminant genomes using BWA-MEM and SAMtools.

### Taxonomic Analyses

All taxonomic analyses were performed at the species level. To account for the compositional nature of the data, a centered-log-ratio (CLR) normalization was applied (Gloor et al. 2017) using the “transform” function of the microbiome R package (V1.12.0; Lahti and Shetty 2018).

#### Oral Composition

The effects of subspecies and sex assignments on oral microbiome composition were assessed using PERMANOVAs performed on Jaccard (Jaccard 1901) and Aitchison distances (Aitchison and Aitchison 1986) using the “adonis’ function of the vegan package (V2.5-7; Oksanen et al. 2011). We ran separate PERMANOVA models for molecularly assigned and museum-recorded sex, since both records were incomplete and conflicted each other for some samples (supplementary table S1, Supplementary Material online). Because of the unequal distribution of host subspecies among the two datasets (“newly generated” vs. “Fellows Yates et al. (2021)”; supplementary fig. S1, Supplementary Material online), a dataset was included in the models before any biological variables. We performed pairwise PERMANOVAs with the “adonis.pair” function of the EcolUtils R package (V0.1; Salazar 2019) using Jaccard and Aitchison distance matrices. Principal coordinate analysis (PCoA) plots were generated with the phyloseq package (V1.34.0, (McMurdie and Holmes 2013)).

#### Alpha Diversity

We estimated species richness using the Chao1 estimator (Chao et al. 2009) and evenness using the Shannon index (Shannon 1948) in phyloseq (V1.34.0, (McMurdie and Holmes 2013)). To assess the effect of the host subspecies on microbial diversity, we ran ANOVA models, transforming Chao1 and Shannon estimates using “sqrt(500-x)” and “exp(x)”, respectively, to ensure the assumptions of normality (Shapiro–Wilk's test on the Studentized residuals) and homogeneity of variance (visual assessment of the fit against the residuals (Quinn and Keough 2002)) were met. For within-factor comparisons, we used Tukey posthoc tests.

#### Identification of Differentially Abundant Taxa

We identified bacterial taxa that significantly differed in abundance between host subspecies using ANCOM-II (Mandal et al. 2015), a method appropriate for compositional data and sparse taxon tables (Kaul et al. 2017). To account for potential dataset-specific contamination (“newly generated” vs. “Fellows Yates et al. (2021)”; supplementary fig. S1, Supplementary Material online), we ran the analysis twice, first identifying microbial taxa that differed in abundance between host subspecies, while accounting for read depth, and then identifying microbial taxa that differed by datasets, while accounting for the host subspecies. Only genera that differed by subspecies, but not by dataset were considered for evaluating microbiome differences among gorilla subspecies. Differentially abundant taxa were identified as those above the 0.9 quantile of the ANCOM W-statistic and Kruskal-Wallace posthoc test (Bonferroni adjusted *P* values < 0.05), or those that were structurally absent from one or more subspecies. Only microbial taxa identified at the genus or species level were considered.

We used the same approach to identify microbial taxa that differed in abundance between mountain and western lowland gorillas. These taxa were then used to explore the effects of altitude, separating Grauer's gorillas into high- and low-altitude populations and performing comparisons using both Jaccard and Aitchison distances in pairwise PERMANOVAs, in which, differences in dataset identity were included as factors.

### Functional Analysis

Using decontaminated metagenomic reads, we characterized the functional capacity of the microbial community with the HUMAnN2 pipeline (Segata et al. 2012; Truong et al. 2015; Franzosa et al. 2018). The abundances of gene families were normalized to copies per million reads and specific functions were regrouped under GO terms (Ashburner et al. 2000; Gene Ontology Consortium 2021). Focusing on the biological processes, we investigated the functional differences in the oral microbiomes of different host subspecies using PERMANOVA on Euclidean distances and ANCOM (for processes present in at least 30% of the samples in a subspecies).

### Metagenome-assembled Genomes

We used the metaWRAP pipeline (V1.3.2; Uritskiy et al. 2018) to construct MAGs from concatenated decontaminated sequencing reads across all samples. Initial assemblies were performed using MEGAHIT (Li et al. 2016), and distinct genomic bins were identified based on the consensus of two different metagenomic binning tools, MaxBin2 (Wu et al. 2016) and metaBAT2 (Kang et al. 2019). The taxonomic identity of each MAG was assigned with GTDB-TK (Chaumeil et al. 2020). The abundance of MAGs in each sample was estimated using salmon (V0.13.1; Patro et al. 2017) and visualized using pheatmap (Kolde 2019). For each assembled and taxonomically identified MAG, we extracted and curated records of isolation sources (supplementary table S7, Supplementary Material online) following an approach described by Madin et al. (2020). This information was used to categorize the isolation sources for taxa into one of the following categories: “contaminant”, “environmental”, “host-associated”, “oral”, or “unknown”.

The evolutionary relationships for select MAGs were evaluated based on amino acid sequence alignments of core genes constructed with PhyloPhlAn (V3.0.2; Segata et al. 2013). A maximum-likelihood phylogeny was created using FastTree (V2.1.10; Price et al. 2010), and visualized with ggtree (V3.1.0; Yu et al. 2017). To determine the presence/absence of genes involved in the nitrate reduction pathway, we used the pangenomic tool Panaroo (Tonkin-Hill et al. 2020) and translated BLAST search (tblastn, V2.11.0; Camacho et al. 2009) using protein sequences obtained from UniProt against MAG contigs.

### Estimating Ecological Variation in Oral Microbiome Composition

Using altitude as a proxy for ecology and diet, we estimated the correlation between altitudinal, geographical, and microbiome distances. Euclidean distance matrices were constructed based on the approximate altitude and geographic coordinates obtained from museum records with the R package vegan. We then performed partial Mantel tests (10,000 permutations) between microbiome distance matrices (Pearson correlation used for Jaccard distances and Spearman's rank coefficient correlation for Aitchison taxonomic and Euclidean functional distances) and altitudinal distances, while accounting for the effect of log-transformed geographical distances in the ade4 R package (1.7–16; Dray and Dufour 2007).

To further investigate the effects of altitude, samples of Grauer's gorillas, the subspecies with the largest altitudinal range (Plumptre et al. 2016), were partitioned into low- (500–1000 masl) and high-altitude (>1000 masl) groups. The contribution of altitude was assessed with PERMANOVAs, accounting for dataset and host subspecies effects.

### Dietary Analysis

Eukaryotic reads in dental calculus were used to identify taxa potentially consumed by gorillas. We removed reads assigned to prokaryotes and viruses from our metagenomes using the “extract_kraken_reads.py” script from KrakenTools and repeated taxonomic classification with Kraken2 using the NCBI “nt” database (accessed September 27, 2021).

#### Preprocessing of the Feature Table

A joint feature table containing species and genus-level assignments was produced using kraken-biom and analyzed with phyloseq alongside a taxonomic matrix, created using the taxize R package. We aggregated all identified taxa to the genus level, and used decontam (V1.10.0; Davis et al. 2018) to remove likely contaminants, as described above for the oral microbiome analyses. We removed genera present in the museum controls or with fewer than 10 reads per sample. Since only a few reference genomes of tropical plants are available and classically used barcoding genes represent only a small part of the genome, accurate taxonomic classification from a sparse metagenomic sample is difficult and can likely only be achieved on genus or family level (Mann et al. 2020). For this reason, and because, despite of our filtering, spurious classifications or contaminants were still present (species that are unlikely to be consumed by gorillas, e.g., mollusks), we focused our analysis on genera previously reported in gorilla diet (Remis et al. 2001; Rogers et al. 2004; Yamagiwa et al. 2005; Rothman et al. 2014; Michel et al. 2022), including all detected genera in the same family. We used ANCOM as described above, to identify differentially abundant dietary taxa while accounting for the number of reads per sample. We estimated DNA damage profiles of a well-represented dietary taxon (*Gallium*) using mapDamage2 (Jónsson et al. 2013), by mapping reads from a highly abundant sample (MTM009, >500 reads) to the closest reference genome. The relationship between microbial and dietary distances was tested using a multiple regression on Aitchison distance matrices using the R package ecodist (Goslee and Urban 2007).

#### Collecting Gorilla Diet Reference Data

We produced a reference dataset of gorilla foods (supplementary table S14, Supplementary Material online) by searching the literature for plant species known to be consumed by the three gorilla subspecies based either on direct observations, analyses of food remains and feces or, more recently, on molecular evidence (Remis et al. 2001; Rogers et al. 2004; Yamagiwa et al. 2005; Rothman et al. 2014; Michel et al. 2022). We retrieved the corresponding taxonomic IDs from NCBI using the taxize R package. For the taxa that had no match in the NCBI Taxonomy database, we manually searched the database and corrected the names that were obviously misspelt (difference of 1–2 characters from the NCBI entry). For the remaining taxa, we manually searched the GBIF species database (Wheeler 2004) and obtained GBIF taxonomic IDs. However, several taxa (26 out of 314) could not be found in either database and, therefore, were excluded from our analysis. The taxonomic IDs obtained from either NCBI or GBIF were used with the “classification” function of taxize to obtain the genus and family name for each taxon.

## Supplementary Material

Supplementary data are available at *Molecular Biology and Evolution* online.

## Supplementary Material

msac263_Supplementary_Data

## Data Availability

Sequencing data generated in this study have been deposited on ENA, under Project Accession Number: PRJEB49638. Scripts for both preprocessing and downstream analysis are available at 10.5281/zenodo.6861585.
