# Supplementary material for: Ecology, Not Host Phylogeny, Shapes the Oral Microbiome in Closely Related Species
Source: Mol Biol Evol. 2022 Dec 6;39(12):msac263. doi: 10.1093/molbev/msac263 (PMC9778846; doi:10.1093/molbev/msac263)
Supplement: msac263_Supplementary_Data [file msac263_supplementary_data.zip › MBE Revisions Supplementary Material.pdf]

Supplementary Methods and Results

Mitochondrial genome analysis for molecular subspecies assignment

We extracted all reads mapping to the gorilla mitochondrial genome, excluding the hypervariable D-loop region (positions 15,447-16,364), with a mapping quality of 30 or higher. For each sample, coverage and total number of reads mapping to the mitochondrial genome were calculated with SAMtools, and a consensus sequence was generated with ANGSD (V0.933; Korneliussen et al., 2014).

We recovered host mitochondrial sequences from 44 of 46 samples, with, on average, 31.2% (0.44-94.2%; Table S2) of the reference mitochondrial genome covered by mapped reads and an average coverage depth of 3.27X (0.01-77.04X). Six samples yielded mitochondrial genomes with completeness >80% and coverage >3X (maximum 94% completeness and 77X coverage, Table S2). Neither sample weight (information available for n=24 samples), sample age (n=36), subspecies identity as obtained from museum records (n=46) nor dataset ("newly generated" versus "Fellows Yates et al. 2021", n=46) had an effect on mtDNA genome completeness (ANOVA, p>0.05).

To confirm subspecies identity of each sample, as available from museum records, we relied on diagnostic sites within the host mitochondrial genome. Using 102 published mitochondrial genomes (Das et al., 2014; Hallast et al., 2016; Hu & Gao, 2016; van der Valk et al., 2018; Xu & Arnason, 1996); Table S11), we identified 403 diagnostic sites (sites that were fixed for different alleles) between western lowland and eastern gorillas and 72 diagnostic sites between mountain and Grauer's gorillas using a custom Python script (10.5281/zenodo.6861585). In most samples, reads mapped to only a small number of diagnostic sites (Table S2). We accepted molecular taxon assignment for samples with reads mapping to at least six diagnostic sites in six separate reads. For six samples, data was insufficient to distinguish between eastern and western gorilla species and for an additional six samples we could not reliably distinguish between mountain and Grauer's gorilla subspecies. In these cases, we accepted the host subspecies identification based on museum records, after consulting records of the collection locality, where available. All but one sample containing a sufficient number of diagnostic sites were successfully assigned to their reported subspecies. The exception was sample MTM010 (museum accession: 631168, Swedish Museum of Natural History - NRM; Table S2), which was confirmed to be a mountain gorilla, in accordance with the museum records, although it was previously reported as a western lowland gorilla based on preliminary genomic information (Fellows Yates et al., 2021).

In many cases, even a few mitochondrial fragments were sufficient to allow subspecies assignment. We could genetically distinguish gorilla species (eastern versus western gorillas) with mitochondrial genome coverage as low as 2.6% and eastern gorilla subspecies (mountain versus Grauer's) with

coverage as low as 6.6%. Grauer's and mountain gorilla mitochondrial genomes could be successfully distinguished despite differing by only 0.5% (72 positions, excluding the hypervariable D-loop region (van der Valk et al. 2018)). This observation supports the usefulness of dental calculus as material for obtaining genetic information about the host (Warinner et al. 2015; Ozga et al. 2016; Warinner 2016; Mann et al. 2018).

Molecular sexing of samples using dental calculus preserved host reads

We used sexassign (Gower et al., 2019) to determine the sex of each individual (Table S1). To this end, we mapped filtered reads, prior to removing host and human reads, to the gorilla reference genome (GCF_000151905.2) and counted reads mapping to the X chromosome and the autosomes. sexassign conducts a likelihood ratio test comparing the observed X-to-autosome ratio to expected ratios for males ($R_X \sim 0.5$) and females ($0.8 > R_X < 1.2$). Of the 46 samples included in the final dataset, sex could be successfully assigned to 38 samples. Molecular sex assignments agreed with museum records for the majority of the samples (n=28), whereas four samples had conflicting assignments. The remaining six samples had no information on sex in the museum records. As these conflicts could be due to errors in museum records, or molecular assignments being impacted by human- or cross-contamination, we ran all analyses that tested for the effects of host sex twice, using only molecular sex assignment and only museum records, respectively.

Supplementary Figures

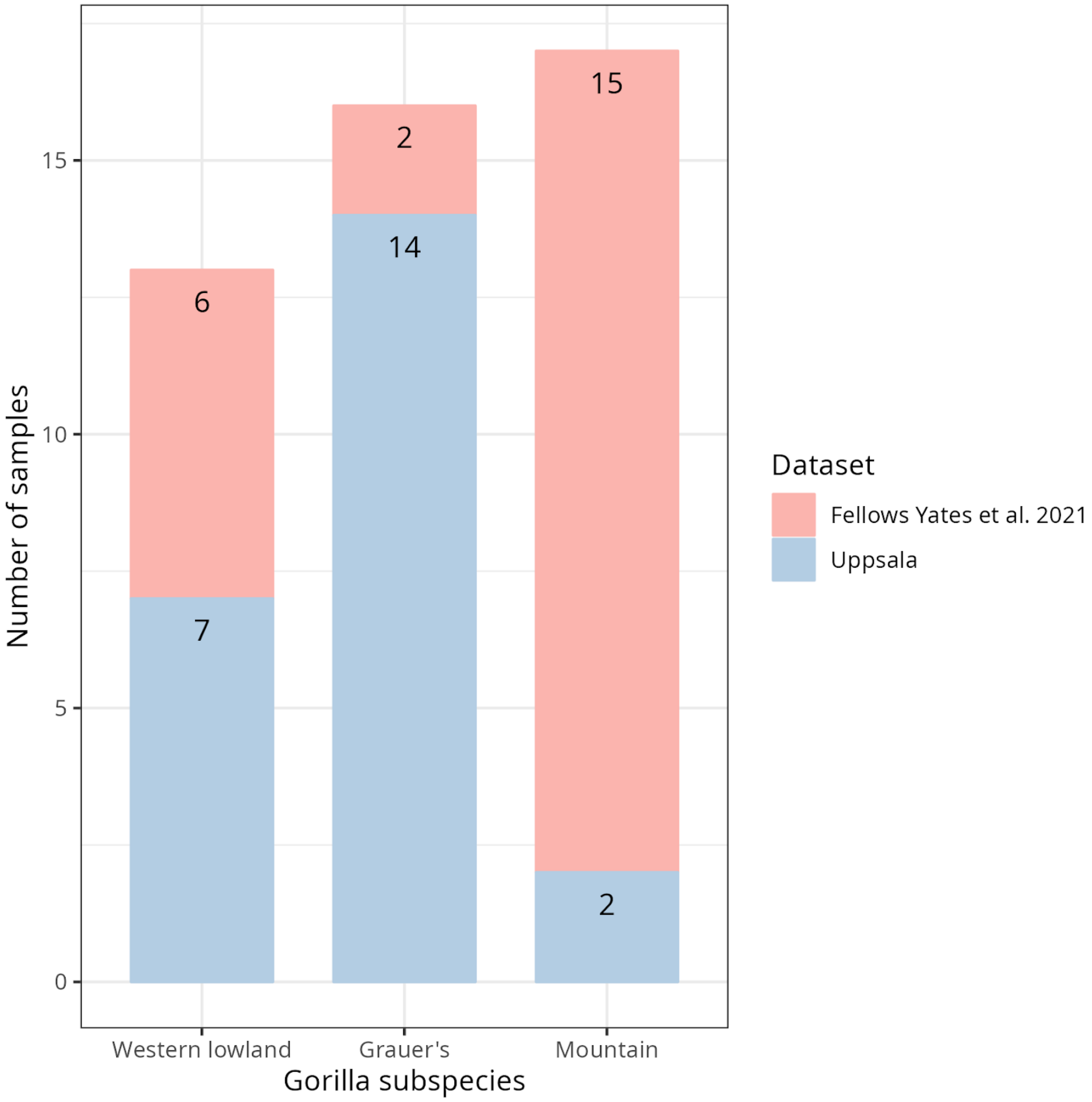

Figure S1. Visual summary of sample contribution to the final dataset. ”Newly generated” data is shown in blue and corresponds to samples processed at Uppsala University, Sweden, consisting of 21 newly sequenced samples and two previously published samples (Brealey et al., 2020). Salmon bars correspond to 23 samples previously published by Fellows Yates et al. (2021) and processed at the Max Planck Institute for the Science of Human History, Jena, Germany.

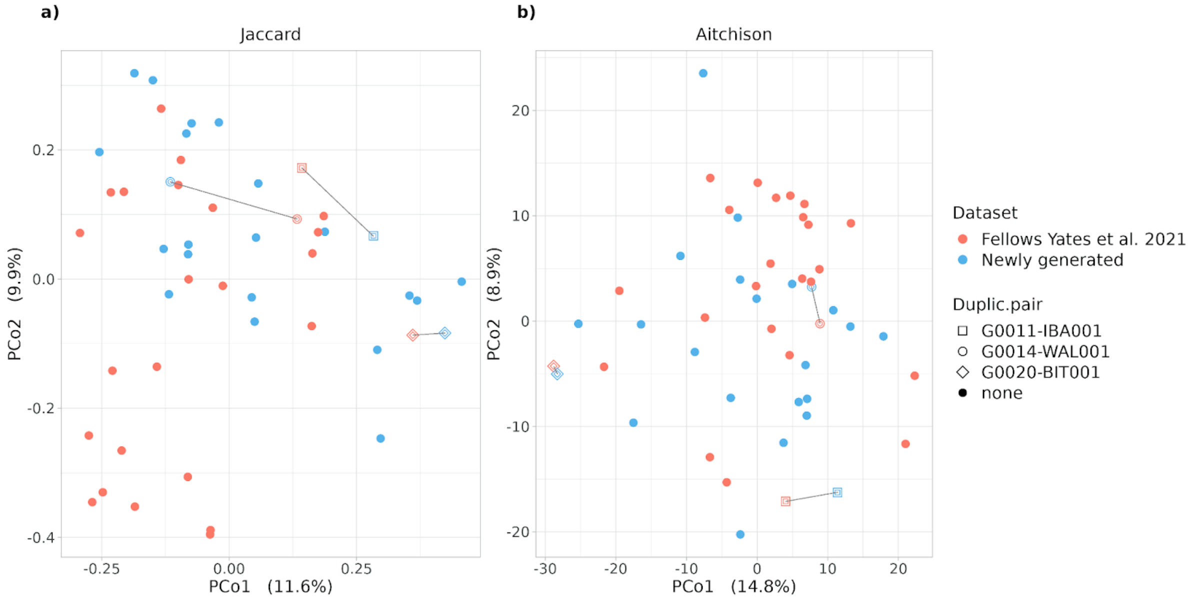

Figure S2. Principal coordinate analysis (PCoA) plots based on **a**) Jaccard distance and **b**) Aitchison distance highlighting pairs of samples that correspond to the same museum specimen but were processed separately in this study and by Fellows Yates et al.(2021). Duplicate pairs are displayed in different shapes and are linked with a grey line. Datasets are displayed in different colours.

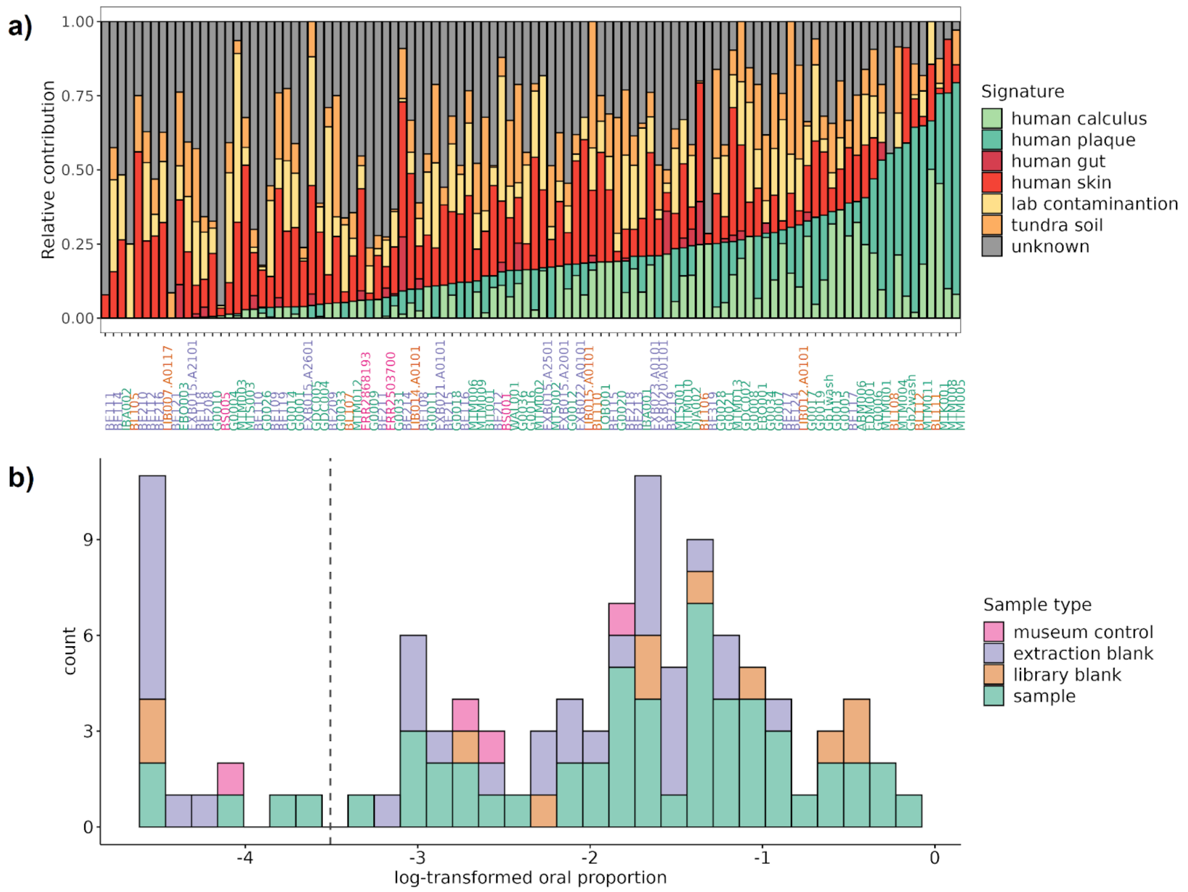

Figure S3. (a) Output of FEAST showing the composition of dental calculus-derived microbial communities partitioned into different putatively contributing environments for each sample. Label colours correspond to sample types, as detailed in b). (b) Histogram showing the log-transformed oral proportion (human calculus and human plaque considered jointly) per sample. Only dental calculus samples with more than 300,000 reads are shown here. Dental calculus samples (coloured in green) with an oral microbiome proportion below 3% (those left of the vertical dashed line) were excluded from analyses. In contrast, all negative controls and museum environment samples were retained. The bars are coloured by sample type: museum controls (pink), extraction blanks (purple), library preparation blanks (orange), specimen samples (green).

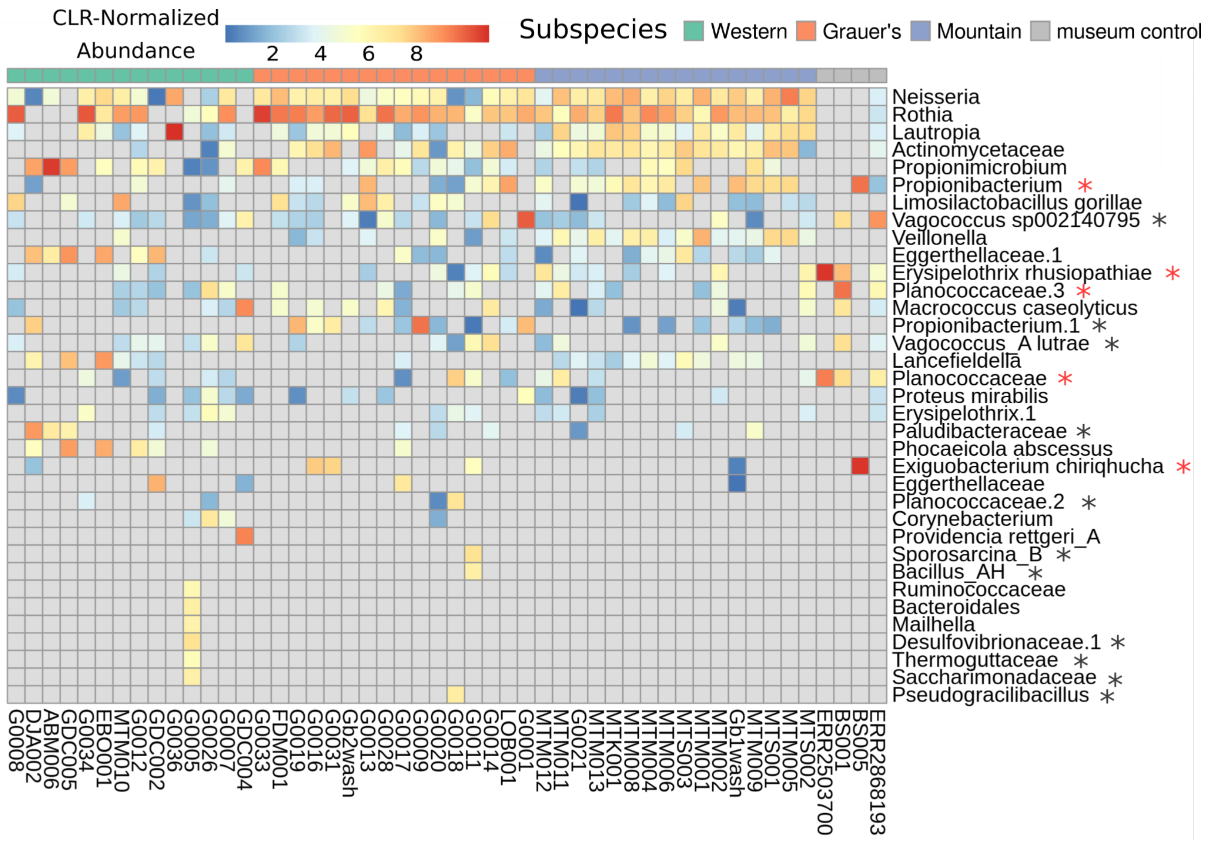

Figure S4. The CLR-normalised abundance of MAGs reconstructed from decontaminated reads. MAG taxonomic identity is provided on the y-axis, and the identity of individual samples is shown on the x-axis. Host subspecies/museum controls are shown on top of the heatmap, including the skull (BS005) and shelf (BS001) swabs, petrous bone (ERR2503700), and skin (ERR286193). MAGs are sorted based on prevalence across all samples, and the absence of a MAG within a sample is shown in grey. Sample G0005 contained many MAGs not found in any other sample, likely due to much deeper sequencing of this sample compared to others (77,307,443 processed reads compared to the mean of 11,365,074 reads per sample). Asterisks denote taxa that are found at higher abundance in at least one museum control compared to any dental calculus samples (red) and taxa likely to be contaminants on the basis of isolation source information (black), as shown in Figure S6.

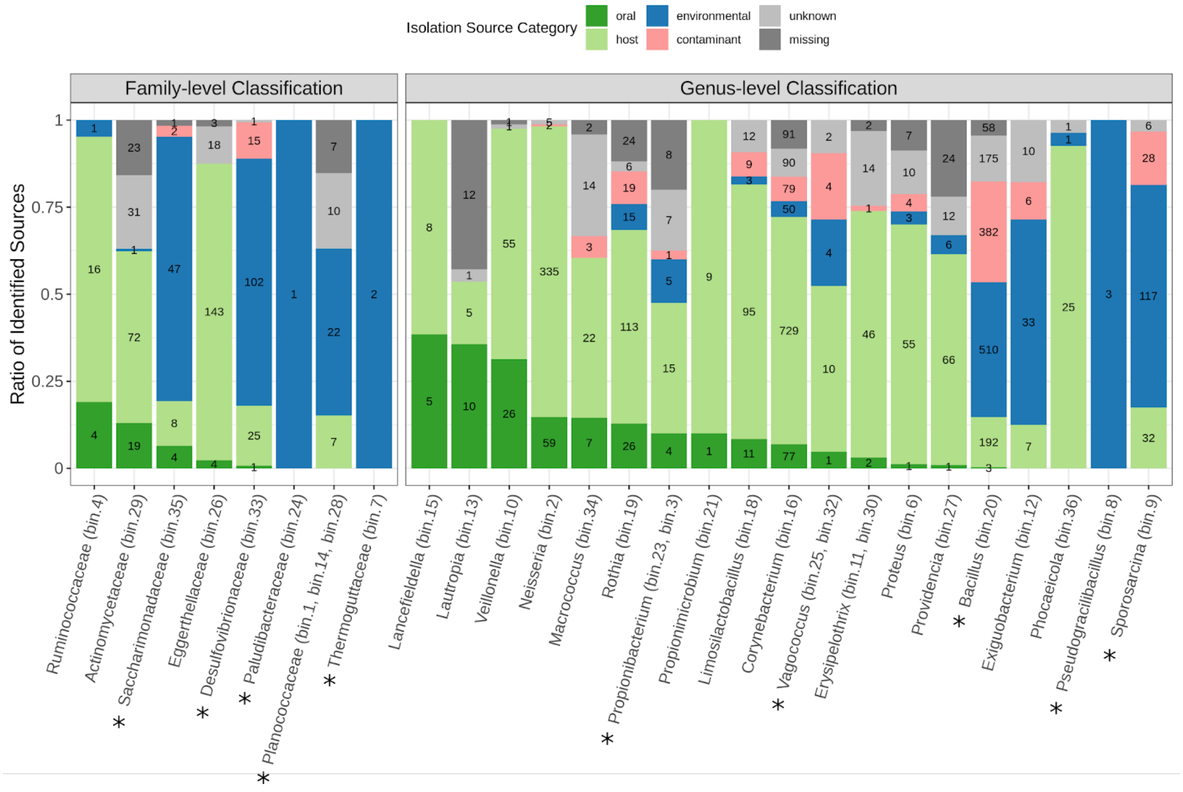

Figure S5. Isolation source information for each MAG taxon was extracted from multiple databases (see Table S8). Broad categories of isolation sources were generated based on the presence of key words. Values within each section of a stacked bar represent the number of unique isolation sources for each category. Taxa with a combined proportion of environmental and contaminate sources greater than 25% were considered as probable contaminants. We included asterisks to denote which MAGs were excluded on the basis of the overrepresentation in environmental/contaminant isolation sources.

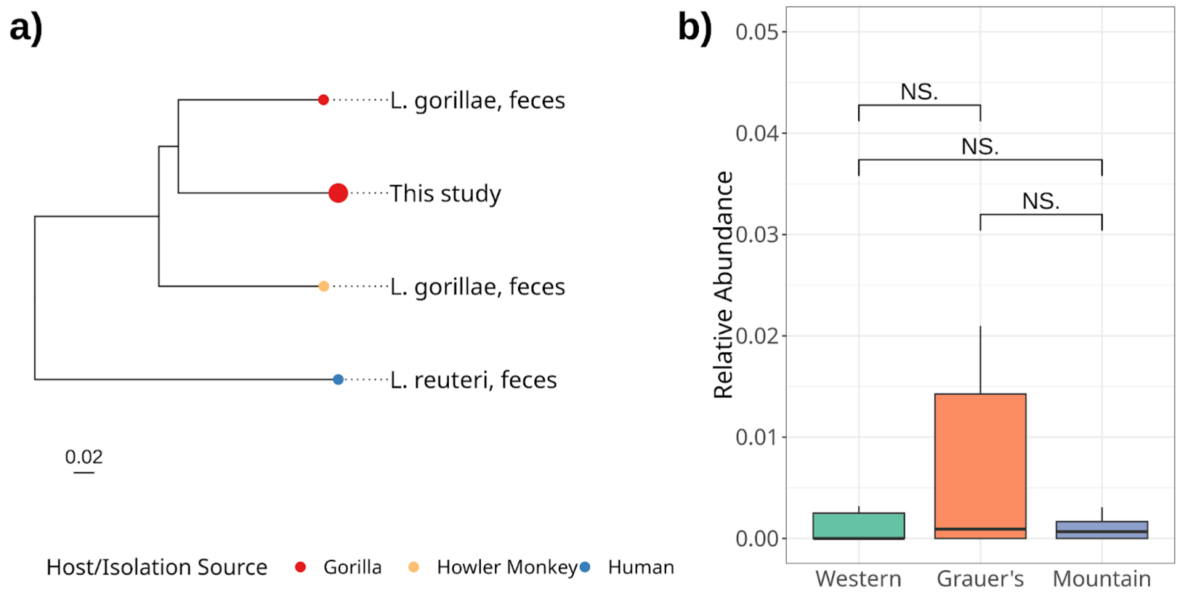

Figure S6. a) Maximum-likelihood phylogeny based on alignment of core gene sequences from *Limosilactobacillus gorilla*. Tips include host species identity and the isolation source for each genome. All nodes have complete support (1.0) after 100 bootstrap replicates, and scale bar units are the number of substitutions per site. **b)** The relative abundances (CLR transformed) between different gorilla subspecies in *Limosilactobacillus gorilla*, with the results of a Wilcoxon test are denoted by brackets above boxplots (NS = not significant).

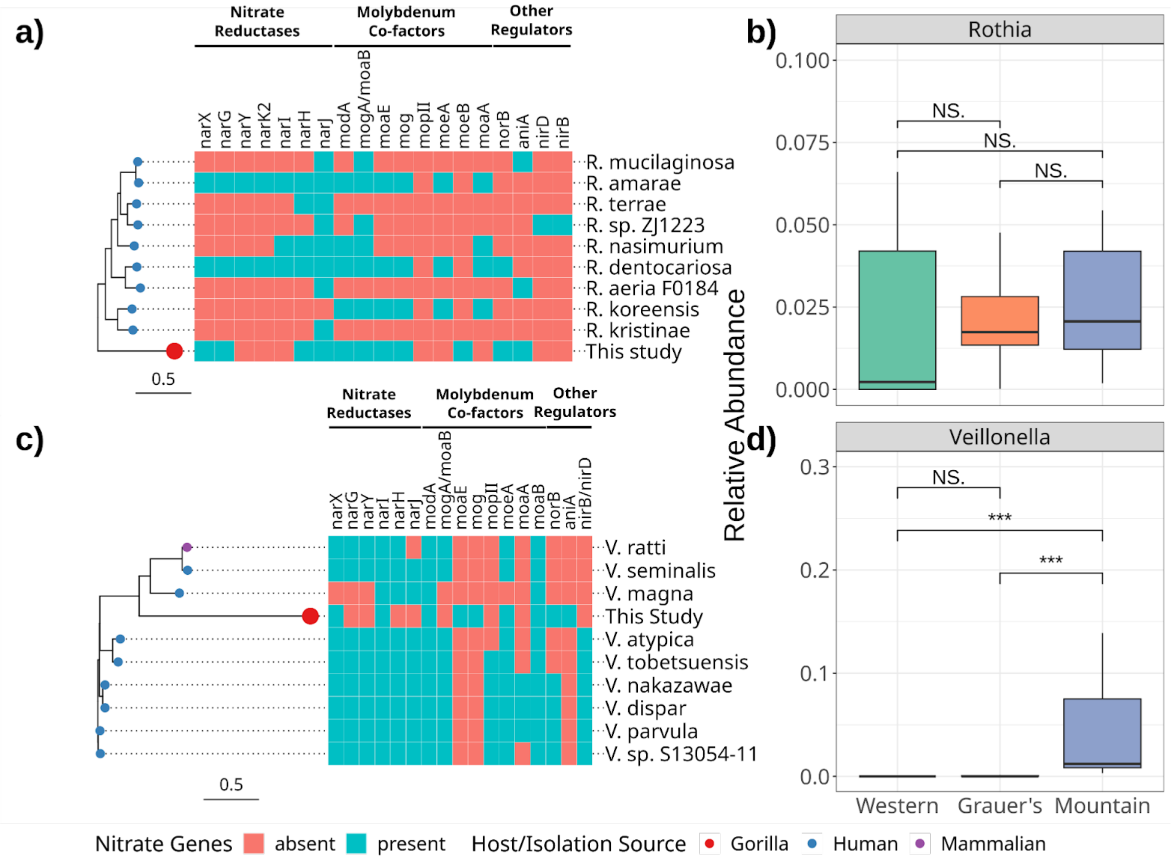

Figure S7. Maximum-likelihood phylogenies based on alignment of core gene sequences from MAGs of **a)** *Rothia* and **c)** *Veillonella*, as well as additional published genomes for each genus. Tip points are coloured by host species identity. Scale bar units are the number of substitutions per site. All nodes have support values of 1 after 100 bootstrap replicates. To the right, the presence of key genes involved in the reduction of nitrate in the oral cavity is shown for each genome. Panels **b)** and **d)** show the relative abundances (CLR transformed) of *Rothia* and *Veillonella* MAGs, respectively, in different gorilla subspecies. The results of a Wilcoxon test are denoted by brackets above boxplots (NS = not significant; *** = p > 0.001).

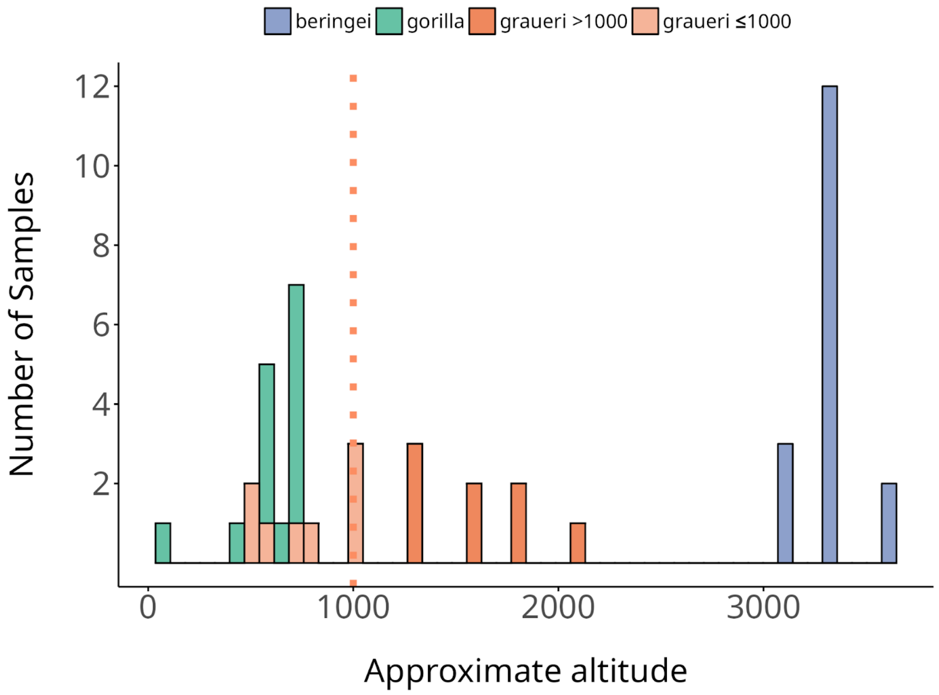

Figure S8. Altitude (in metres above sea level, masl) of each gorilla specimen used in this study. Dotted line at 1000 masl denotes the separation between Grauer's gorilla altitude groups (low altitude ≤1000 masl, and high altitude >1000 masl).

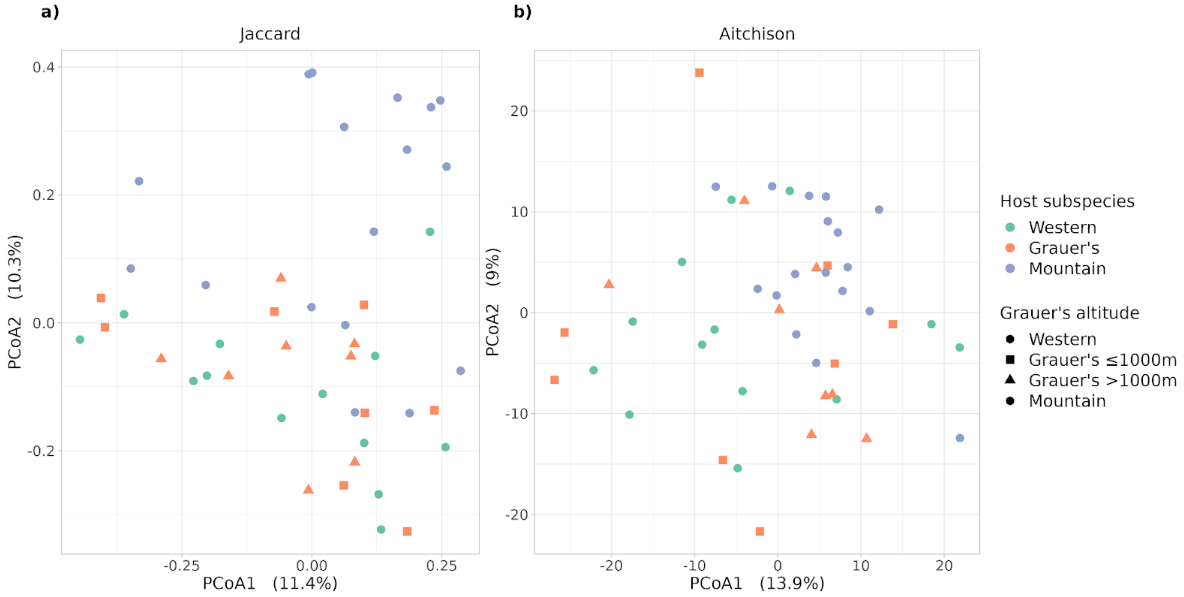

Figure S9. Principal coordinate analysis (PCoA) plots highlighting the effect of altitude on the oral microbiomes of Grauer's gorillas based on a) Jaccard distance and b) Aitchison distance. Host subspecies is displayed in different colours. Grauer's gorillas from low altitudes are shown as squares and from high altitudes as triangles.

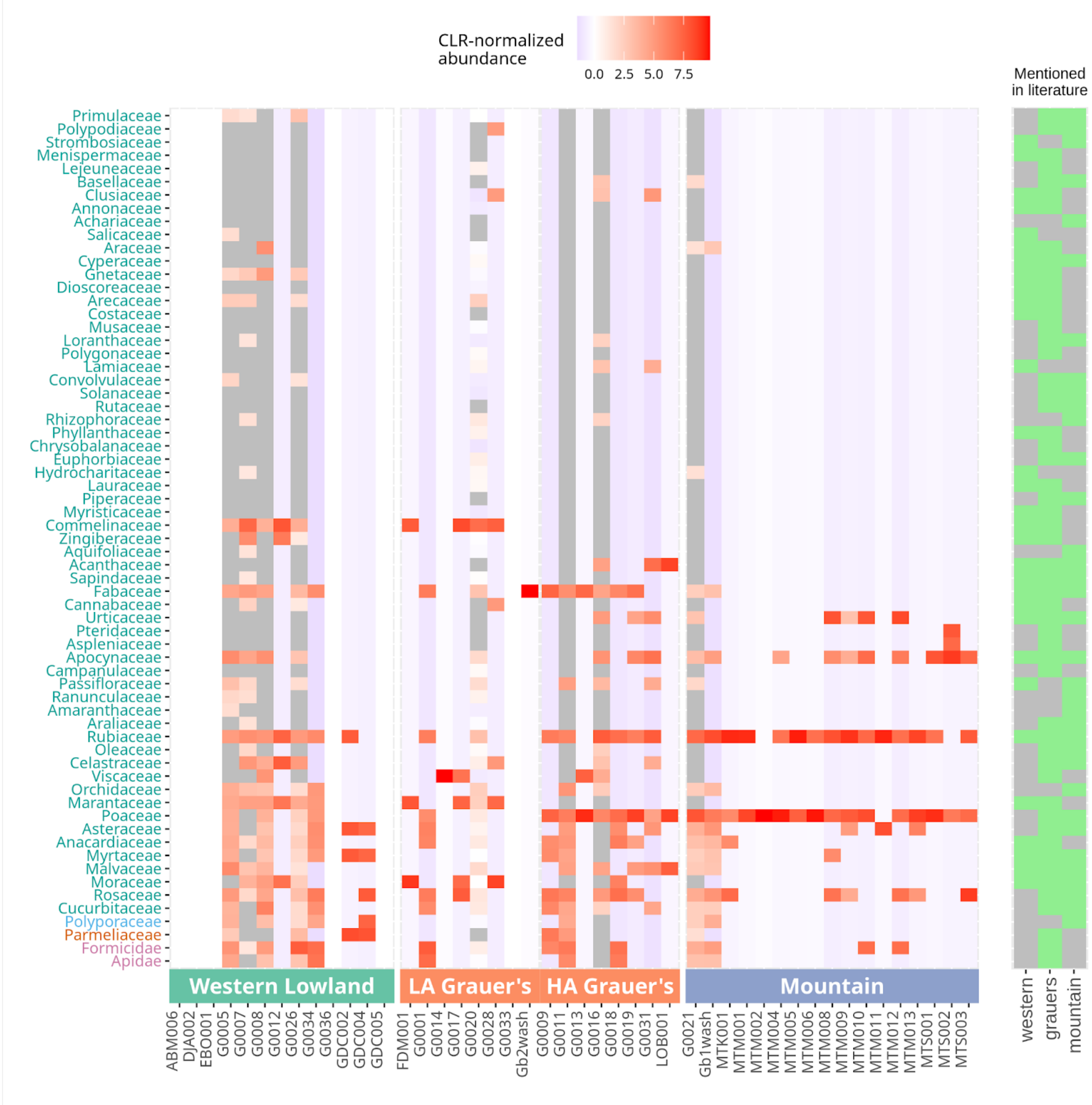

Figure S10. Heatmap based on CLR-normalised abundance of eukaryotic families that are known to be consumed by gorillas. Labels of the y axis are coloured based on the phylum (green: higher plants; blue: Basidiomycota; red: Ascomycota; pink: arthropods). The bar on the right indicates if the family has been listed as part of the diet of each gorilla subspecies (grey: absent; green: present).

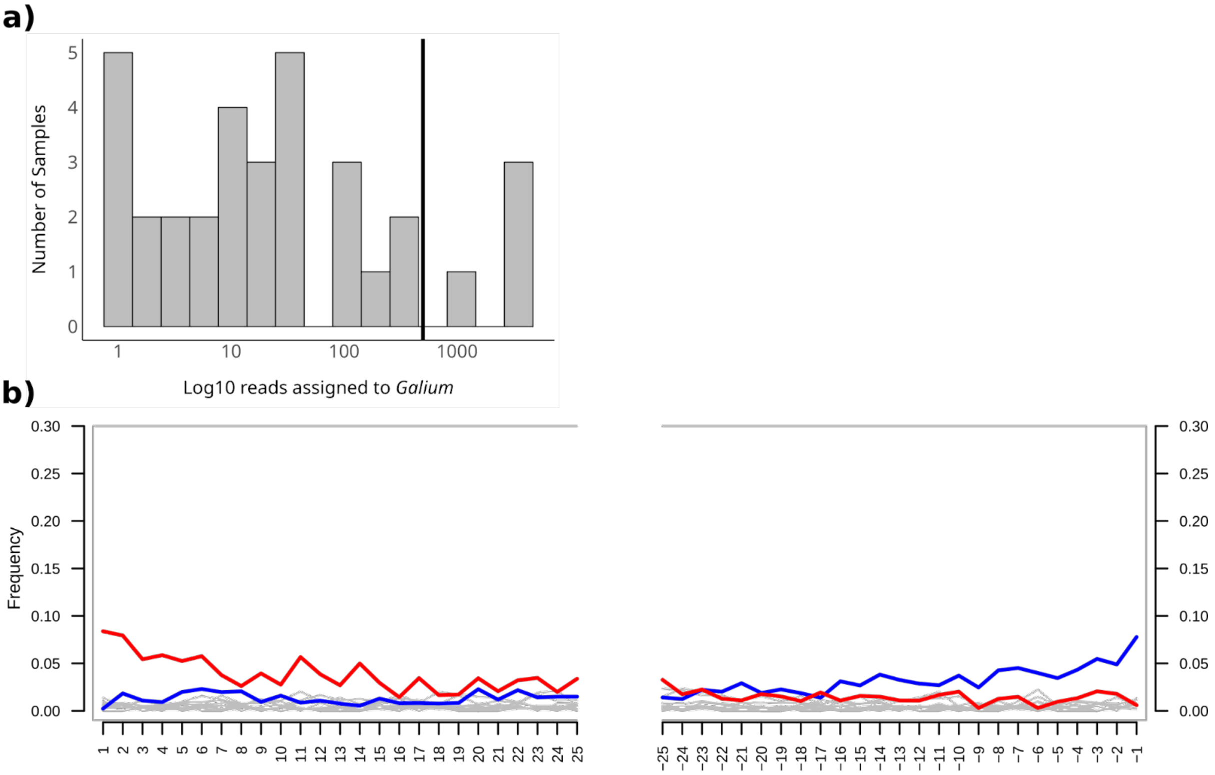

Figure S11. Authentication of dietary components. **a)** Summary of read numbers assigned to *Galium* in the study samples, with a vertical line at 500 reads, a minimum identified for informative investigation of post-mortem DNA damage (Mann et al., 2020). **b)** Rates of nucleotide misincorporation in 5' and 3' ends of reads from sample MTM009 that mapped to the *Galium porrigens var. tenue* reference genome (GCA_012274505.1). Red line= C-to-T substitutions, blue line= G-to-A substitutions, grey line= all other substitutions.

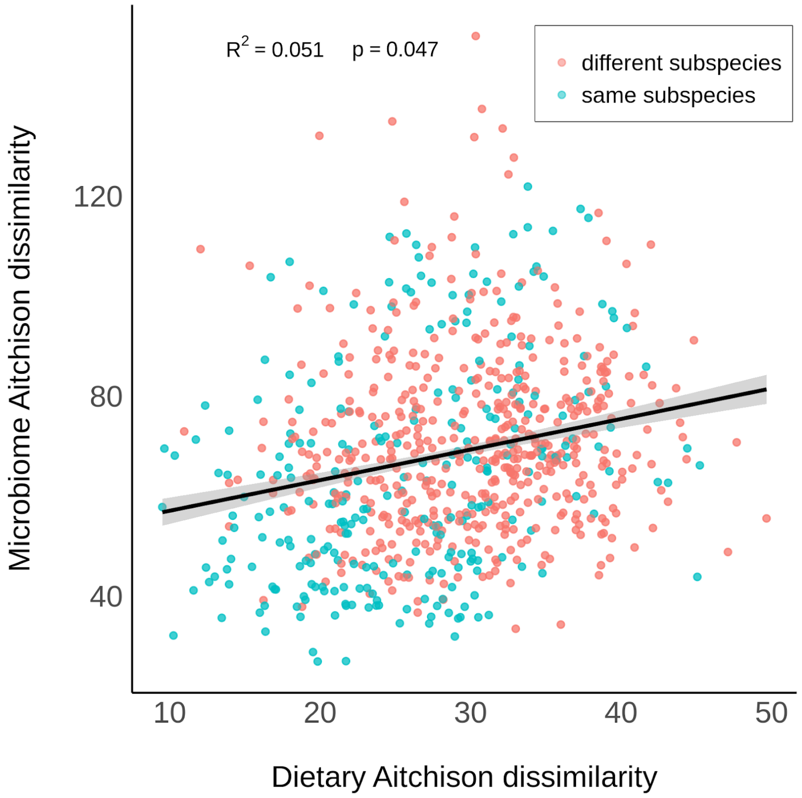

Figure S12. The relationship between the microbial and dietary components in gorilla dental calculus is demonstrated using multiple regression (MRM) of their respective distance matrices (based on Aitchison distances). The MRM was run for 10,000 permutations and the reported p-value is the result of a pseudo-t-test on these iterations.

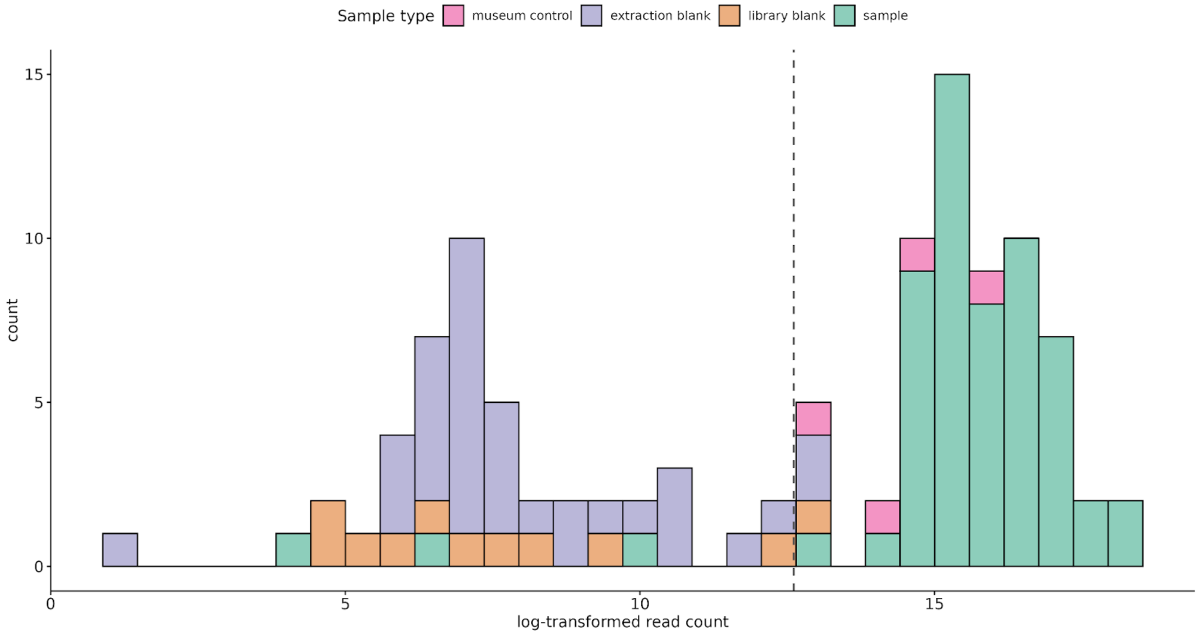

Figure S13. Histogram showing the read count (log-transformed) after pre-processing for all samples that were subjected to taxonomic classification. The bars are coloured by sample type: museum controls (pink), extraction blanks (purple), library preparation blanks (orange), specimen samples (green). Dental calculus samples (green) containing less than 300,000 reads (those left of the vertical dashed line) were excluded.

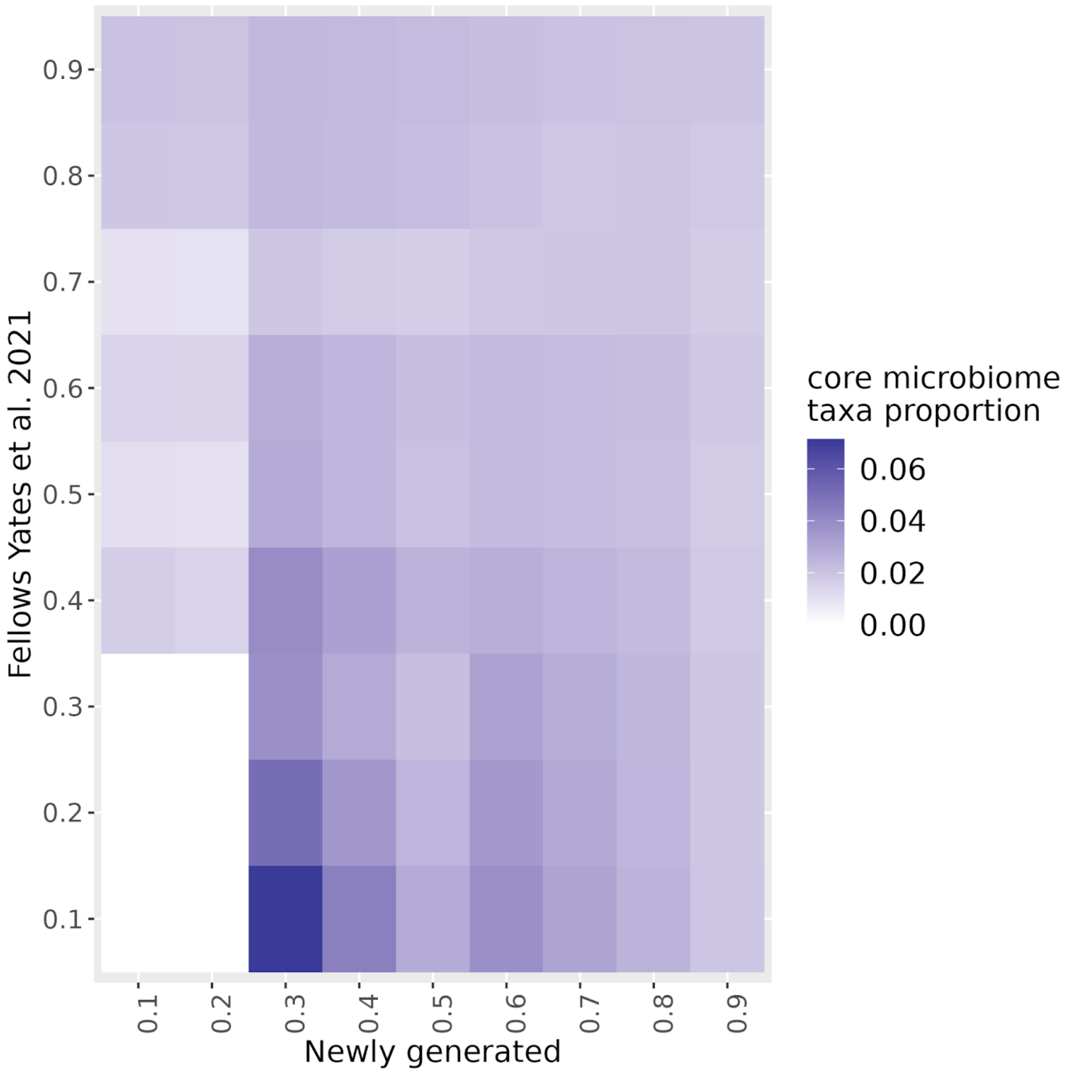

Figure S14. Heatmap showing the proportion of oral taxa among the total taxa identified as contaminants with the R packaged decontam using different thresholds for the newly generated (x-axis) and Fellows Yates et al.(2021) (y-axis) datasets.

Supplementary References

Brealey, J. C., Leitão, H. G., van der Valk, T., Xu, W., Bougiouri, K., Dalén, L., & Guschanski, K. (2020). Dental Calculus as a Tool to Study the Evolution of the Mammalian Oral Microbiome. *Molecular Biology and Evolution*, *37*(10), 3003–3022.

Das, R., Hergenrother, S. D., Soto-Calderón, I. D., Dew, J. L., Anthony, N. M., & Jensen-Seaman, M. I. (2014). Complete mitochondrial genome sequence of the Eastern gorilla (Gorilla beringei) and implications for african ape biogeography. *The Journal of Heredity*, *105*(6), 752–761.

Fellows Yates, J. A., Velsko, I. M., Aron, F., Posth, C., Hofman, C. A., Austin, R. M., Parker, C. E., Mann, A. E., Nägele, K., Arthur, K. W., Arthur, J. W., Bauer, C. C., Crevecoeur, I., Cupillard, C., Curtis, M. C., Dalén, L., Díaz-Zorita Bonilla, M., Díez Fernández-Lomana, J. C., Drucker, D. G., … Warinner, C. (2021). The evolution and changing ecology of the African hominid oral microbiome. *Proceedings of the National Academy of Sciences of the United States of America*, *118*(20). https://doi.org/10.1073/pnas.2021655118

Gower, G., Fenderson, L. E., Salis, A. T., Helgen, K. M., van Loenen, A. L., Heiniger, H., Hofman-Kamińska, E., Kowalczyk, R., Mitchell, K. J., Llamas, B., & Cooper, A. (2019). Widespread male sex bias in mammal fossil and museum collections. *Proceedings of the National Academy of Sciences of the United States of America*, *116*(38), 19019–19024.

Hallast, P., Maisano Delser, P., Batini, C., Zadik, D., Rocchi, M., Schempp, W., Tyler-Smith, C., & Jobling, M. A. (2016). Great ape Y Chromosome and mitochondrial DNA phylogenies reflect subspecies structure and patterns of mating and dispersal. *Genome Research*, *26*(4), 427–439.

Hu, X.-D., & Gao, L.-Z. (2016). The complete mitochondrial genome of eastern lowland gorilla, Gorilla beringei graueri, and comparative mitochondrial genomics of Gorilla species. *Mitochondrial DNA. Part A, DNA Mapping, Sequencing, and Analysis*, *27*(2), 1484–1485.

Korneliussen, T. S., Albrechtsen, A., & Nielsen, R. (2014). ANGSD: Analysis of Next Generation Sequencing Data. *BMC Bioinformatics*, *15*, 356.

Mann, A. E., Fellows Yates, J. A., Fagernäs, Z., Austin, R. M., Nelson, E. A., & Hofman, C. A. (2020). Do I have something in my teeth? The trouble with genetic analyses of diet from archaeological dental calculus. *Quaternary International: The Journal of the International Union for Quaternary Research*. https://doi.org/10.1016/j.quaint.2020.11.019

van der Valk, T., Sandoval-Castellanos, E., Caillaud, D., Ngobobo, U., Binyinyi, E., Nishuli, R., Stoinski, T., Gilissen, E., Sonet, G., Semal, P., Kalthoff, D. C., Dalén, L., & Guschanski, K. (2018). Significant loss of mitochondrial diversity within the last century due to extinction of peripheral populations in eastern gorillas. *Scientific Reports*, *8*(1), 6551.

Xu, X., & Arnason, U. (1996). A complete sequence of the mitochondrial genome of the western lowland gorilla. *Molecular Biology and Evolution*, *13*(5), 691–698.
